# Qualitative and Quantitative Study of Glycosphingolipids in Human Milk and Bovine Milk Using High Performance Liquid Chromatography–Data-Dependent Acquisition–Mass Spectrometry

**DOI:** 10.3390/molecules25174024

**Published:** 2020-09-03

**Authors:** Lin Ma, Bertram Y. Fong, Alastair K. H. MacGibbon, Gillian Norris

**Affiliations:** 1 Fonterra Research and Development Centre, Dairy Farm Road, Private Bag 11029, Palmerston North 4442, New Zealand; Bertram.Fong@fonterra.com (B.Y.F.); alastair.macgibbon@fonterra.com (A.K.H.M.); 2 School of Fundamental Sciences, Massey University, Private Bag 11222, Palmerston North 4442, New Zealand; G.Norris@massey.ac.nz

**Keywords:** high performance liquid chromatography–tandem mass spectrometry, glucosylceramide, galactosylceramide, lactosylceramide, bovine milk, human milk, MFGM Lipid 100

## Abstract

Cerebrosides (Crb; including glucosylceramide and galactosylceramide) and lactosylceramide (LacCer) are structurally complex lipids found in many eukaryotic cell membranes, where they play important roles in cell growth, apoptosis, cell recognition and signaling. They are also found in mammalian milk as part of the milk fat globule membrane (MFGM), making milk an important dietary component for the rapidly growing infant. This study reports the development of a robust analytical method for the identification and characterization of 44 Crb and 23 LacCer molecular species in milk, using high performance liquid chromatography–tandem mass spectrometry in data-dependent acquisition mode. For the first time, it also compares the distributions of these species in human and bovine milks, a commercial MFGM-enriched dairy ingredient (MFGM Lipid 100) and commercial standards purified from bovine milk. A method for quantifying Crb and LacCer in milk using mass spectrometry in neutral loss scan mode was developed and validated for human milk, bovine milk and MFGM Lipid 100. Human milk was found to contain approximately 9.9–17.4 µg Crb/mL and 1.3–3.0 µg LacCer/mL, whereas bovine milk (pooled milk from a Friesian herd) contained 9.8–12.0 and 14.3–16.2 µg/mL of these lipids, respectively. The process used to produce MFGM Lipid 100 was shown to have enriched these components to 448 and 1036 µg/g, respectively. No significant changes in the concentrations of both Crb and LacCer were observed during lactation.

## 1. Introduction

**Figure 1:**
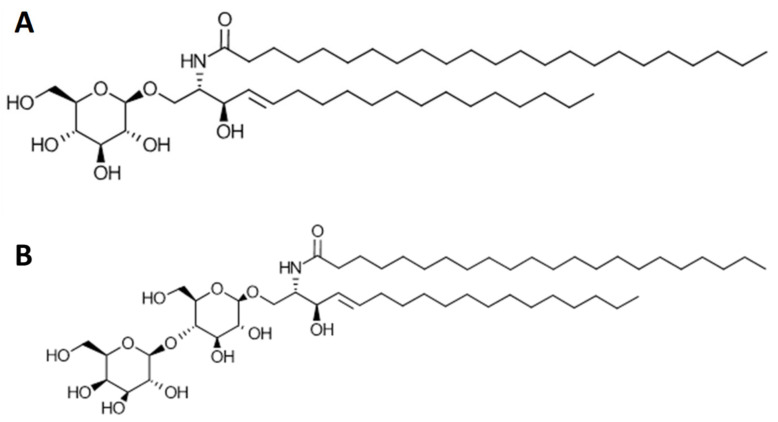
Schematic structures of glucosylceramide (GluCer) (d18:1/23:0, **A**) and lactosylceramide (LacCer) (d18:1/22:0, **B**).

Neutral glycosphingolipids (GSLs) are complex lipids that belong to the sphingolipid family (with lipid classification codes of SP05 and SP [1]). They are amphiphilic molecules consisting of a polar head group attached to a non-polar ceramide backbone. The polar head groups typically contain sugar residues. For instance, glucosylceramide (GluCer), galactosylceramide (GalCer) and lactosylceramide (LacCer) contain a glucose, a galactose and a lactose polar head group, respectively. The hydrophobic ceramide backbone is composed of a fatty acid chain and a sphingoid base. Both the fatty acid chain and the sphingoid base of the ceramide can vary in length (the number of carbon atoms) and degree of unsaturation (the number of double bonds), resulting in an even greater diversity of molecular species within each GSL class. Figure 1 shows the schematic structures of a GluCer (upper panel) and a LacCer (lower panel) with their d-values (the sum of the carbon atoms in the sphingoid base and the fatty acid moiety and the number of double bonds within them), where “d” indicates a dihydroxy compound. GSLs and ceramides are important precursors for the biosynthesis of a wide range of sphingolipids such as gangliosides and sphingomyelin [2,3].

Sphingolipids, including GluCer and LacCer, are believed to play important roles in signal transduction, cell recognition, cell adhesion, cell growth and protein trafficking [4,5,6]. Their involvement in pathogen defense and infection has also been reported [7]. Moreover, GluCer, GalCer and LacCer are important components of the neural system and play a role in brain development, in both the fetus and new-born animals including humans [6,8,9]. GSLs, together with sphingomyelin, contribute to neonatal cognition [10,11,12].

Although GluCer and LacCer are found primarily in cellular membranes [6,13,14], they are also present in bovine milk, predominantly in the milk fat globule membrane (MFGM) [15,16]. However, very limited information related to their composition and concentration in both human milk and bovine milk is available. Whereas GluCer has been reported to be the major GSL in bovine and ovine milks [17,18], GalCer has been reported to be the major GSL in human milk [18,19]. However, despite these reports, little analytical evidence that distinguishes GluCer from GalCer in human and bovine milks has been published. Because of the very similar structures and isobaric masses of these two GSL molecules, it is difficult to separate them by liquid chromatography and to characterize them using mass spectrometry without additional treatment. In this study, where the two GSLs (GluCer and GalCer) are isobaric, and therefore, cannot be distinguished, they are collectively referred to as cerebrosides (Crbs).

The concentrations of Crbs and LacCer in bovine milk and human milk have previously been reported using high performance liquid chromatography with ultraviolet detection (HPLC-UV) [18]. There is, however, a paucity of information on the diversity of the molecular species found in these natural secretions. The aim of this study was therefore to develop a robust one-step LC-MS method to determine and compare the compositions and concentrations of Crb and LacCer in complex matrices such as human and bovine milks and in products made from the latter. The ability to reliably determine the composition of milk products in such detail will enable manufacturers to address any potential differences between the concentrations of these important components in bovine-milk-based infant formula and human milk and, on the basis of the data obtained, to ‘humanize’ infant formula to a greater extent.

## 2. Results

### 2.1. Characterization of Crbs and LacCer

#### 2.1.1. Commercial Standards

**Figure 2:**
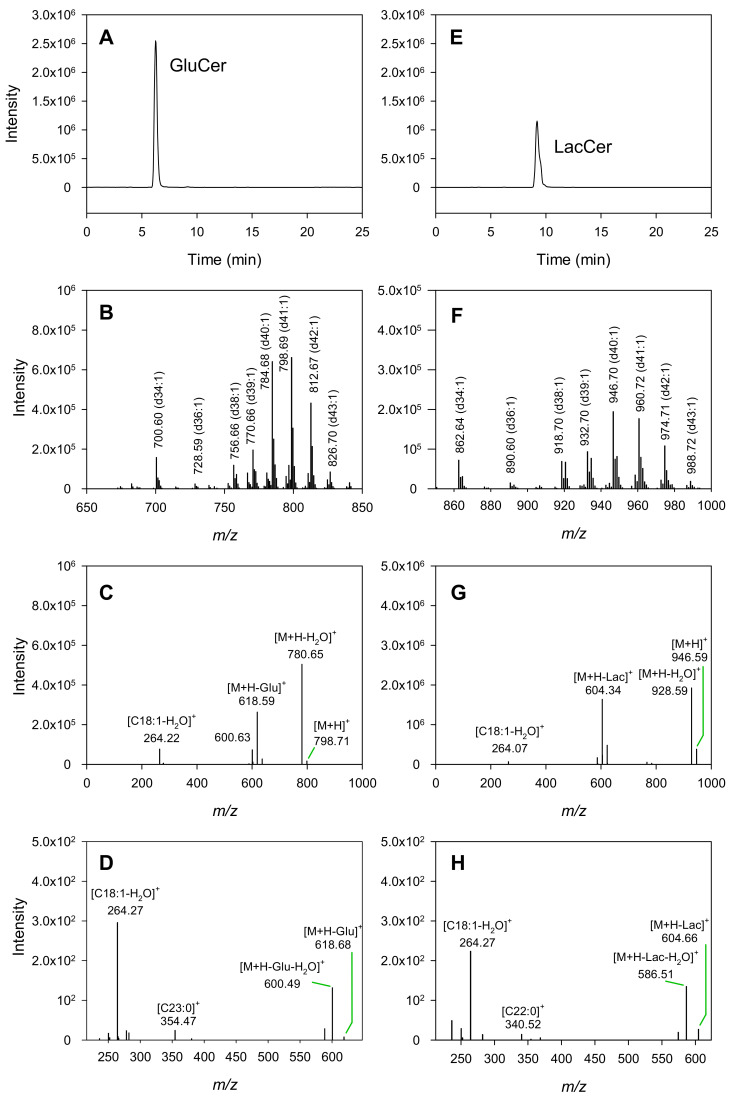
Typical mass spectra showing the total ion counts (TICs) of GluCer (**A**) and LacCer (**E**), commercial standards purified from buttermilk, separated using hydrophilic interaction liquid chromatography (HILIC) and detected in positive ion mode as protonated molecules. Corresponding mass spectra are presented in (**B**) and (**F**), respectively. The tandem mass spectrometry (MS^2^) fragmentation of the GluCer *m/z* 798.71 amu ion (**C**) and the LacCer *m/z* 946.59 amu ion (**G**) are shown, followed by the corresponding three-stage mass spectrometry (MS^3^) fragmentations of the daughter ions with *m/z* 618.68 amu (**D**) and *m/z* 604.66 amu (**H**), respectively.

Typical mass spectroscopy total ion count (TIC) chromatograms of GluCer and LacCer standards separated by hydrophilic interaction liquid chromatography (HILIC) are presented in Figure 2A,E, respectively. Their corresponding mass spectra are shown in Figure 2B,F. Typical tandem mass spectrometry (MS^2^) fragmentation of selected GluCer (*m/z* 798.69 amu) and LacCer (*m/z* 946.70 amu) ions are shown in Figure 2C,G. The corresponding three-stage MS (MS^3^) fragmentations of selected daughter ions (*m/z* 618.59 amu and *m/z* 604.34 amu) are shown in Figure 2D,H. The accumulated MS, MS^2^ and MS^3^ data were used to identify the different sphingoid bases and fatty acid moieties of the molecular species observed in both the GluCer standard and the LacCer standard. For example, the MS^2^ spectrum of the GluCer molecular species *m/z* 798.69 amu (Figure 2B) shows numerous product ions (Figure 2C), including one with *m/z* 264.22 amu, which is characteristic of the *m/z* for sphingosine d18:1 (mono-dehydrated), and another with *m/z* 618.59 amu, which can be assigned as the deglycosylated parent ([M+H–glucose]^+^). When further fragmented, the 618.59 amu ion produced an MS^3^ ion of *m/z* 354.47 amu, corresponding to a C23:0 fatty acid (Figure 2D). Using these combined MS data, the GluCer molecular species *m/z* 798.69 amu was tentatively assigned as d18:1/23:0. The same procedure was used to identify all the other GluCer peaks in the spectrum. Furthermore, adjacent pairs of ions observed under the Crb peak (Figure 2B), such as *m/z* 784.68 and 798.69 amu, and *m/z* 700.60 and 728.59 amu, differed by either 14 amu or 28 amu, suggesting that they were structurally different by one or two CH_2_ groups on either the LCB or the fatty acid moiety.

A similar strategy was used to characterize LacCer. For example, the MS^2^ spectrum of the ion with *m/z* 946.70 amu (Figure 2F) produced daughter ions of *m/z* 604.34 amu, identified as the delactosylated parent ([M+H–lactose]^+^), and *m/z* 264.07 amu, characteristic of sphingosine d18:1 (Figure 2G). When the daughter ion at 604.34 amu was further fragmented, the MS^3^ ion at *m/z* 340.52 amu was identified as the amide form of the fatty acid C22:0 (Figure 2H). From this evidence, the ion at *m/z* 946.70 amu was tentatively identified as LacCer d18:1/22:0. All other LacCer molecular species were identified using the same procedure.

The monoisotopic masses for all the GluCer and LacCer molecular species present in the commercial standards and their tentative identifications according to their observed *m/z* are summarized in Tables S1 and S2 from Supplementary Materials, respectively. The identification is based on comparison of the observed *m/z* with the calculated *m/z* for the sphingoid or long chain base (LCB) and fatty amide fragments. When the fatty amide fragment was not observed, the proposed tentative identification was based on the difference between the parent *m/z* and the sphingoid base *m/z*. The proportions of the different LCBs and fatty acid moieties found in these standards are summarized in Table 1 and Table 2.

#### 2.1.2. Human Milk, Bovine Milk and MFGM Lipid 100

**Figure 3:**
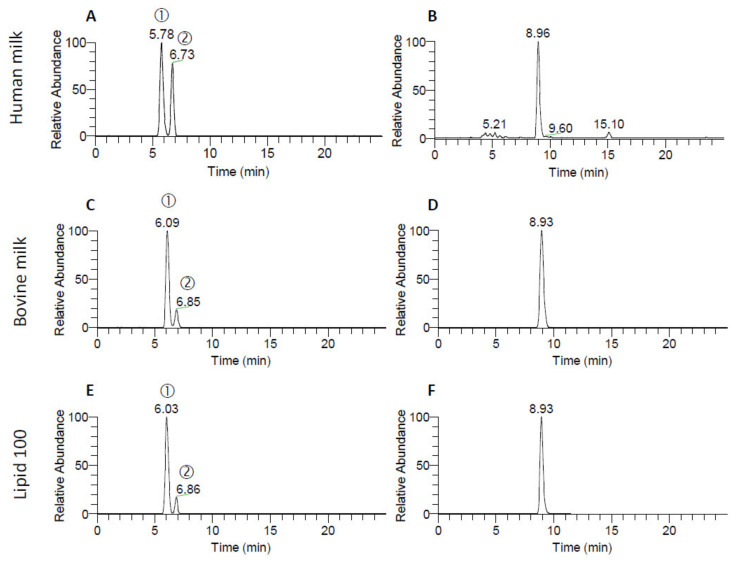
TICs of the HILIC separation of Crb (**A**) and LacCer (**B**) for human milk, bovine milk (**C**,**D**) and MFGM Lipid 100 (**E**,**F**). The first and second Crb peaks in each sample (**A**,**C**,**E**) are labeled as ① and ②, respectively.

The TIC traces for human milk, bovine milk and a commercial MFGM-enriched lipid ingredient (MFGM Lipid 100) separated by HILIC are presented in Figure 3. Surprisingly, twin peaks (labeled as ① and ②) were observed for the Crbs in all samples (Figure 3A,C,E) but not in the standards (Figure 2). Human milk contained relatively higher concentrations of the second peak compared to bovine milk and MFGM Lipid 100. In contrast, only one peak was observed for LacCer, consistent with the standard. Thus, the identification of the second Crb peak was essential for correct quantification.

**Figure 4:**
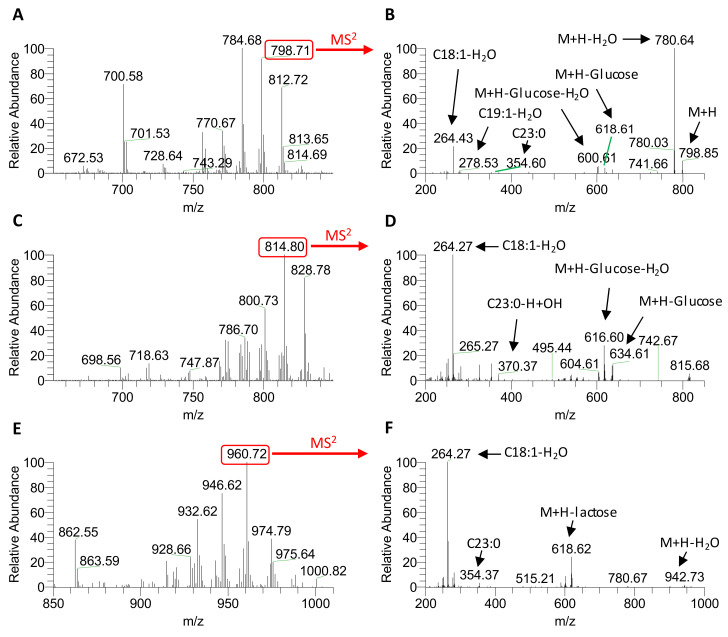
Mass spectra (positive mode) of Crb peak 1 (**A**), Crb peak 2 (**C**) and LacCer (**E**) from bovine milk and the MS^2^ spectra of the 798.73 amu ion from Crb peak 1 (**B**), the 814.56 amu ion from Crb peak 2 (**D**) and the 960.73 amu ion from LacCer (**F**).

The identification of the Crb (peaks 1 and 2) and LacCer molecular species was carried out using MS and MS^2^ data-dependent acquisition (DDA). Typical mass spectra for Crb peaks 1 and 2 and LacCer in bovine milk are shown in Figure 4A,C,E, respectively. Fragmentation spectra of the Crb *m/z* 798.71 amu ion from peak 1, the Crb *m/z* 814.80 amu ion from peak 2 and the LacCer *m/z* 960.72 amu ion are shown in Figure 4B,D,F. Ions with masses equal to those of the LCBs and fatty acids with the loss of a water molecule, a monosaccharide or a disaccharide were observed in the MS^2^ spectra of the respective molecular ions.

The molecular species found in Crb (peak 1) and LacCer were tentatively assigned based on the fragments using the same principles that were used to characterize the standards. However, the molecular ions observed in the mass spectrum of the Crb peak 2 were different to those observed in the mass spectrum of Crb peak 1 (Figure 4C). It is worthwhile to mention that the product ion *m/z* 370.37 amu in the MS^2^ spectrum of the ion *m/z* 814.80 amu from Crb peak 2 (Figure 4D) was identified as the hydroxylated fatty acid C23:0. Non-hydroxylated C23:0 has a theoretical molecular mass of 354.6, making the increase in mass of 16 Da for the observed ion most likely to have been a consequence of the addition of a hydroxyl group. The remaining ions under Crb peak 2 of both milk types and MFGM Lipid 100 were also characterized using combined MS and MS^2^ data. Again, these daughter ions represented species that were predominantly hydroxylated, explaining their longer retention times compared to their non-hydroxylated equivalents, which eluted earlier. The presence of ions differing by 14 amu mirrored the observations made for peak 1.

**Figure 5:**
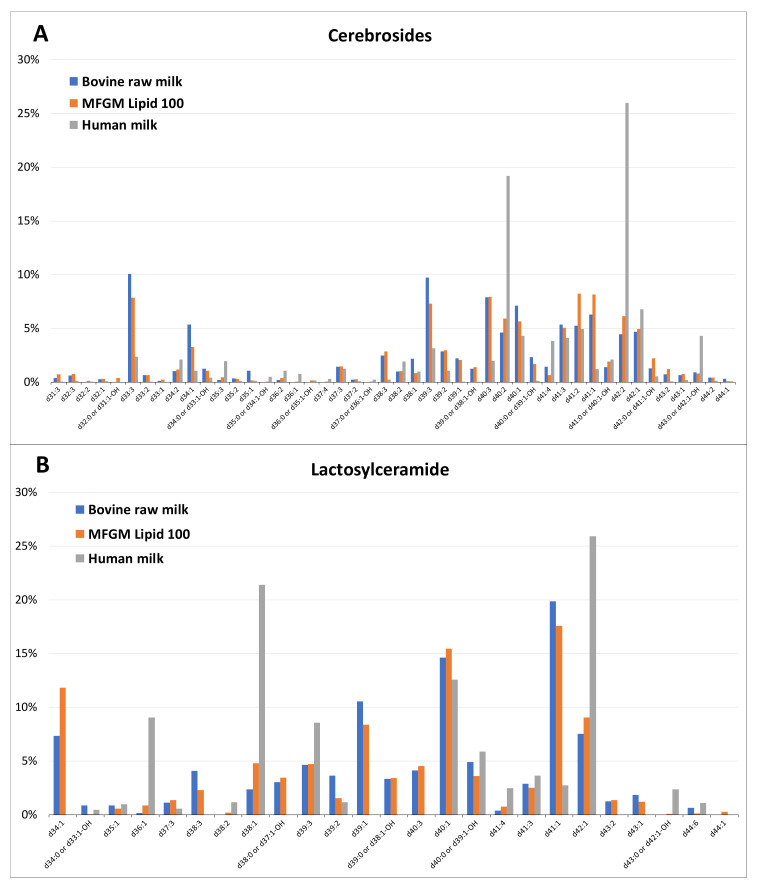
Proportional distribution of the major molecular species (indicated as the “d” number) of Crbs (**A**) and LacCer (**B**) in bovine milk, MFGM Lipid 100 and human milk.

Using this MS strategy, all the Crb and LacCer molecular ions observed were characterized and their distributions (as percentages of intensity) are presented in Figure 5 for both milk types and MFGM Lipid 100. A detailed summary of the fragmentation of all observed GSL molecular species, along with their proposed identifications, is listed in the Supplementary Materials (Tables S3 and S4 for human milk, Tables S5 and S6 for bovine milk and Tables S7 and S8 for MFGM Lipid 100). Summaries of all the different LCBs and fatty acid moieties identified in proportion for all samples are shown in Table 1 and Table 2, respectively.

**Figure 6:**
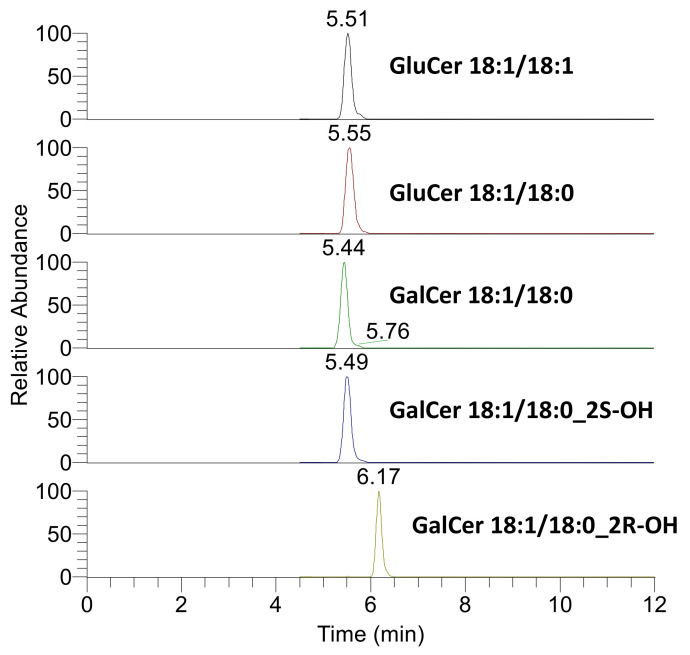
Chromatograms of five synthetic Crb standards characterized by different fatty acyl chains, different monosaccharide head groups and different stereochemistries.

To further determine the identity of the second Crb peak, samples of the synthetic standards GluCer d18:1/18:1, GluCer d18:1/18:0, GalCer d18:1/18:0, GalCer d18:1/18:0 (2R–OH) and GalCer d18:1/18:0 (2S–OH) were individually injected using a fixed-volume sample loop. The resulting chromatograms are shown in Figure 6. There was no significant difference in retention time for most of these molecules, including d18:1/18:0 (2S–OH). Only d18:1/18:0 (2R–OH) (where the hydroxyl group is in the R configuration) had a different retention time and this molecule eluted with the same relative retention time as peak 2.

### 2.2. Impact of GSL Composition on MS Ionization

**Figure 7:**
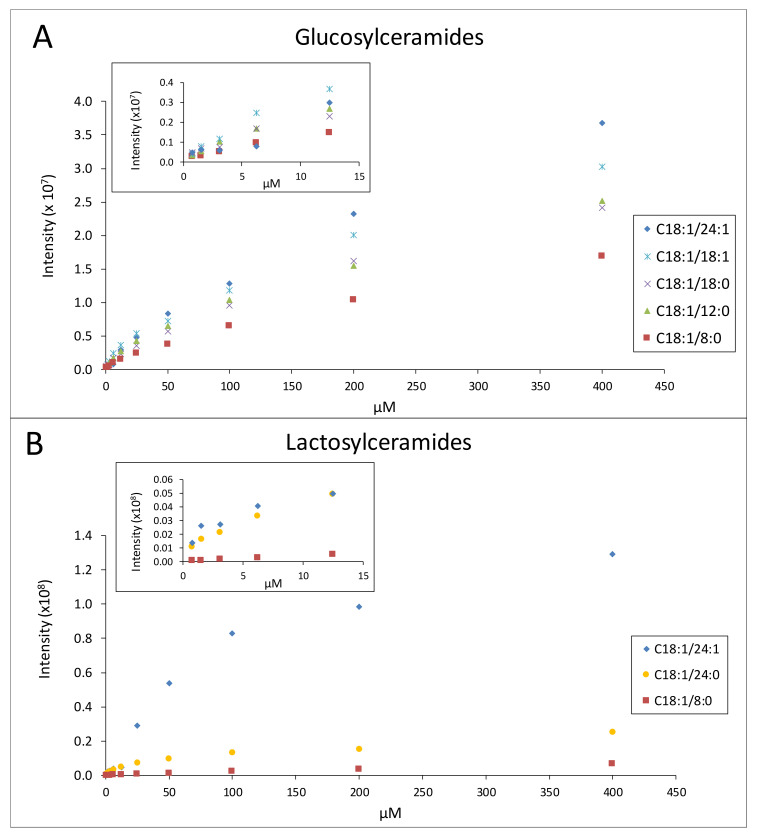
MS response of five different species of GluCer (**A**) and three different species of LacCer (**B**).

The MS responses for five GluCer molecular species and three LacCer molecular species with concentrations ranging from 0.78 to 400 µM are presented in Figure 7. The results show that the GSL ionization efficiencies increased as the fatty acid chain length and/or the degree of unsaturation increased. In contrast, a comparison of the ionization efficiencies of GluCer 18:1/18:0 and GalCer 18:1/18:0 showed that the Crb polar head group did not have any impact on the ionization efficiency (Supplementary Materials, Table S9).

### 2.3. Quantification of Crbs and LacCer

#### 2.3.1. Validation

The limit of detection (LOD) was determined to be 0.2 µg/mL per sample for GluCer and 0.4 µg/mL per sample for LacCer; the limit of quantification (LOQ) was determined to be 0.8 µg/mL per sample for GluCer and 1.6 µg/mL per sample for LacCer. The linear response range was between 0.08 and 20 µg/mL for GluCer and between 0.16 and 20 µg/mL for LacCer.

Spike recovery involved spiking approximately 100% and 200% of the endogenous levels of both GluCer and LacCer into human milk, bovine milk and MFGM Lipid 100. The recovery rates are shown in Table 3.

The reproducibility of these measurements, as defined by the coefficients of variation (CVs), are shown in Table 4. The high CV for LacCer measured in human milk was due to the low concentrations (close to the LOQ) present.

#### 2.3.2. Crb And Laccer Concentrations in Dairy Samples and Human Milk at Different Time Points During Lactation

The averaged human milk Crb and LacCer concentrations determined over 12 months of lactation from Chinese and Malaysian mothers are presented in Table 4. A single human milk sample was used as a quality control to assess the reproducibility of the method and was not included as part of the lactation data analysis. The Crb and LacCer concentrations in three bovine milk samples and a MFGM Lipid 100 sample were measured in duplicate over 3 days. Their concentrations ± standard deviations are shown in Table 4.

#### 2.3.3. Lactational Changes in Crb and LacCer

**Figure 8:**
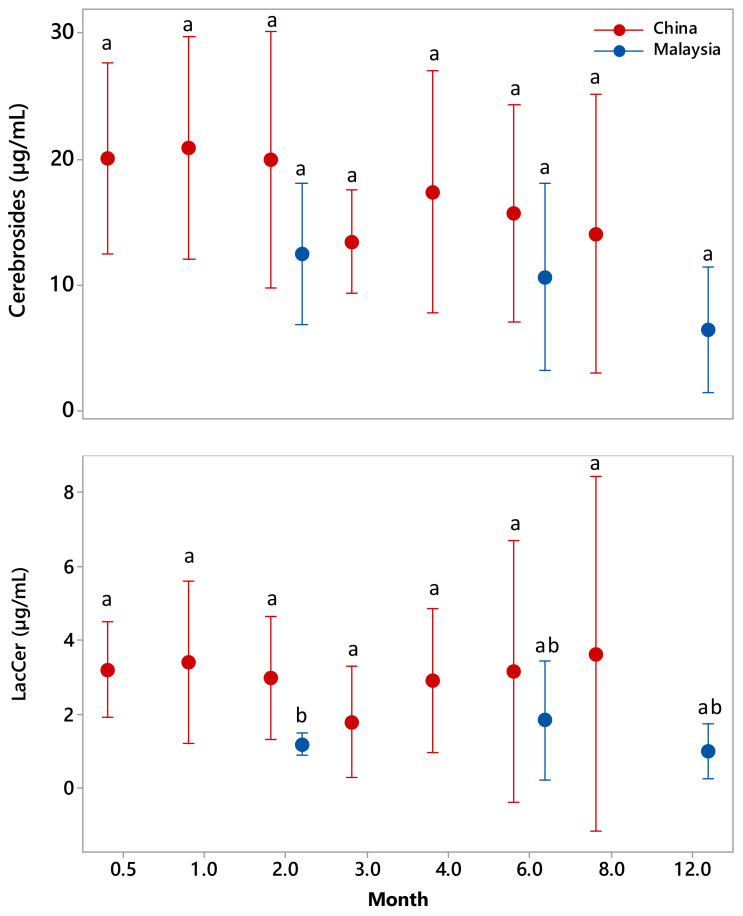
Lactational changes in the concentration of Crb (top panel) and LacCer (bottom panel) in human milk from cohorts of Chinese and Malaysian mothers. The results are at 95% confidence. Data with the same letter indicate no statistically significant difference by one-way analysis of variance or two-sample t-test analysis (*p* > 0.05).

Crb and LacCer concentrations in the milk from five Chinese donors across seven time points (0.5, 1, 2, 3, 4, 6 and 8 months post-partum) and five Malaysian donors across three time points (2, 6 and 12 months post-partum) were measured using the MS methods developed in this study. The averaged Crb and LacCer concentrations for each cohort are shown in Table 4. The changes in the Crb and LacCer concentrations across the lactational time points are shown in Figure 8. Statistical analysis (one-way analysis of variance) showed that there was no significant difference in the concentrations of either Crb or LacCer within each cohort over the time period tested and only a single significant difference at 2 months (*p* < 0.05) between the two cohorts when analyzed using the two-sample t-test.

## 3. Discussion

### 3.1. Characterization of GSLs

#### 3.1.1. LCB and Fatty Acid Moieties in the Purified Standards

Buttermilk-sourced commercial standards were shown to contain 19 GluCer and 14 LacCer molecular species, as shown in Tables S1 and S2 from Supplementary Materials. They were structurally characterized using MS^2^ and MS^3^ fragmentations. MS^2^ fragmentation typically generated product ions that included the dehydrated or de-glycosylated protonated parent, and a dehydrated sphingoid base. It was clear from the MS results that the GluCer and LacCer moieties identified in these commercial standards had similar acyl fatty acid compositions (Figure 2B,F), with similar relative distributions. The Δ *m/z* between GluCer and LacCer with same “d” number is 162, because of the addition of a hexose to glucose to form lactose. These observations comply with the fact that GluCer is the precursor of LacCer in the de novo metabolic pathway. Fragmentation of the dehydrated sphingoid bases showed that the most common sphingoid backbones in these purified standards were dehydrated d18:1 sphingosine (approximately 35%, Table 1) and dehydrated d16:1 sphingoid base (7–18%), although other backbones, such as the d16:0 (11–18%) and d18:0 (18–24%) sphinganines and d17:1 sphingoid base, were also found in some cases (Tables S1 and S2)). The presence of multiple LCBs and fatty acid fragments for some parent ions indicated that different isobaric molecular species were present. This was not surprising given that these standards were purified from a bovine milk source. Similar findings were also reported by Karlsson et al. [15].

In contrast, C22:0, C23:0 and C24:0 were the most common fatty acyl moieties found in the standards, comprising approximately 64% of the total GluCer moieties and 77% of the total LacCer moieties (Table 2). Other long chain fatty acids such as C16:0 and C18:0 and very long chain fatty acids such as C25:0 were more common than the minority monounsaturated fatty acids such as C16:1 and C24:1.

#### 3.1.2. LCB and Fatty Acid Moieties in Human Milk, Bovine Milk and MFGM Lipid 100

Two adjacent peaks (peak 1 and peak 2) were observed for Crb when extracts from human milk (Figure 3A), bovine milk (Figure 3C) and MFGM Lipid 100 (Figure 3E) were subjected to HILIC. HPLC-MS/MS analysis in DDA mode showed that peak 2 of Crb predominantly contained a fatty acyl moiety that was hydroxylated. This identification was confirmed using a GalCer d18:1/18:0–OH standard in which a fatty acyl moiety was hydroxylated in an R-configuration. This is consistent with previous reports of naturally occurring R-hydroxylated fatty acids in animals and in higher plants [20]. No further work to determine the configuration of the hydroxyl group of the hydroxylated Crb present in the samples was carried out. They were not detected in the commercial buttermilk-sourced GluCer standard as they were probably lost during the purification and enrichment process.

Neither the accumulated MS^2^ data nor the retention time data provided enough information to confirm the type of sugar residue (either a glucose or a galactose) associated with the Crb. As such, in this work, the mono-hexosyl-ceramides are reported as Crb without distinction between GluCer and GalCer. However, the historical literature typically refers to the Crb in bovine milk as GluCer [17,18,21], whereas GalCer is reported to be dominant in human milk [18,19]. In contrast, LacCer was identified by both its retention time and accumulated MS^2^ information.

In total, we were able to identify 41, 38 and 44 different Crb molecular species (including hydroxylated species) and 16, 17 and 22 LacCer molecular species in human milk, bovine milk and MFGM Lipid 100, respectively. A diverse mixture of sphingoid bases was observed in the standards and samples analyzed. Of these, the sphingosine d18:1 was the main base detected in Crb, with a higher incidence (54%) in human milk compared to that in dairy samples (39% in bovine milk and 31% in MFGM Lipid 100; Table 1). A similar result was also observed for LacCer, in which d18:1 was the major sphingosine base in human milk (47%) compared with bovine milk (29%) and MFGM Lipid 100 (36%) (Table 1).

Although the major backbone in both Crb and LacCer was d18:1 in both types of milk and MFGM Lipid 100, the sphingoid bases d16:0, d16:1, d17:0, d17:1 and d18:0 were also identified. Interestingly, in human milk LacCer, d19:1 was found to be the second most dominant sphingoid base, which was not the case in bovine milk or its associated product. Other minor sphingoid bases detected included d15:0 and d15:1 and some very long chain bases including d20:1, d22:2 and d24:1. (Table 1). We also found the unusual sphingoid base d21:4 in LacCer, albeit at the limits of detection; it was not observed in any Crb sample.

To our knowledge, this is the first study to investigate the GSL sphingoid base composition in human milk and, as such, no comparison with other reported data could be made. An interesting finding is that the sphingoid base structure in human milk GSLs appears to be more diverse than that reported for sphingomyelin, a phospholipid that is present in high concentrations in human milk. The most common sphingoid base in sphingomyelin has been reported to be d18:1 (sphingosine, 83.6 ± 3.5%), followed by d18:2 (4,8-sphingadienine, 7.2 ± 1.9%) and t18:0 (4-hydroxysphinganine, 5.7 ± 0.7%), with very minor levels of t18:1 (4-hydroxy-8-sphingenine, 0.6–1%) [22].

The diversity in the sphingolipid bases observed for bovine milk in this study was consistent with that reported by Morrison and Hay [21], where d18:1 was the major sphingosine base (~48.1%) in Crb, followed by d16:1 at 10.6% and d17:1 at 6.6%. The dominant fatty acid moieties were saturated, with the carbon chain length varying between C16:0 and C25:0 (or between d34 and d43) (Figure 5 and Table 2) and were present in significantly higher proportions in LacCer (59–74%) than in Crb (29–31%). In contrast, polyunsaturated fatty acids were present in approximately similar proportions in Crb (23–32%) and LacCer (21–36%) in both human and bovine milk and in MFGM Lipid 100 (Table 2). Hydroxylated fatty acids were, however, only observed in Crb (16–20%) and not in LacCer (Table 2). In general, the fatty acid moieties of both Crb and LacCer in dairy samples were much more diverse than those in the standards, despite being purified from the same raw material. This could have been due only to some unknown selectivity that occurred during their purification.

It was interesting to find that the distribution of molecular species was quite distinct between human milk and bovine milk Crbs. In bovine milk, there appeared to be two clusters of molecular species: a small cluster with long chain fatty acids (d33–d36), whereas most were characterized by long chain fatty acids (d38–d40, Figure 7A). In contrast, although human milk Crbs had a higher proportion of very long chain fatty acids, especially d42:2 and d40:2, long chain fatty acids, d38–d40, were also present in lower proportions, although they were often more unsaturated (Figure 7A). Similar results have been previously reported for human milk [19], suggesting that very long chain fatty acids may play a developmental role in human infants. Furthermore, in contrast to the results reported by Bourlieu et al. [23], we found more variation in the species of Crb in the dairy samples compared with their precursor ceramides in buttermilk and butter serum, especially in polyunsaturated species (Figure 7A). However, the molecular profiles of LacCer in the dairy samples were quite similar, except for the significantly higher proportion of d39:1. Although these differences probably reflect the different diets of the animals and extraction, they may also reflect differences in Crb biosynthesis that have evolved because they provide some advantage to the animal and its progeny.

Very few hydroxylated fatty acids (no more than 3%) were identified in LacCer, either in the standards or in the samples analyzed in this study, in contrast to Crb (Table 2). Although similar results were reported by Bouhours and Bouhours for human milk [19], Morrison and Hay [21] reported that bovine milk LacCer contained a diverse range of hydroxylated fatty acids with C16:0–OH, C22:0–OH, C23–OH and C24:0–OH making up 10.5, 15.4, 26.9 and 29.5% of the total detected. However, the proportion of hydroxylated fatty acids to total fatty acids was not provided, making direct comparison difficult. It is possible that the discrepancy may lie with the analytical methods used. Morrison and Hay [21] used a combination of thin layer chromatography, chemical derivation and gas chromatography for analysis, as opposed to the HPLC-MS/MS methods used in this study. The distribution of LacCer molecular species between human milk and bovine milk was also quite distinct, with human milk containing a higher proportion of even-numbered and longer-chain-length fatty acids. Whereas bovine milk contains a reasonable spread of long to very long fatty acids (d38 to d43), human milk is dominated by two very long chain fatty acids, d40:1 and d42:1, and one long chain moiety d38:1 (Figure 5B). Furthermore, only 23 different species were observed for LacCer compared with the 44 species for Crb. Given that GluCer is the biosynthetic precursor of LacCer [24], this was totally unexpected and difficult to rationalize. The differences in the Crb and LacCer distributions in human milk and bovine milk could possibly be because GluCer is the dominant Crb present in bovine milk [18], whereas GalCer is dominant in human milk [19]. Alternatively, it is possible that the mammary gland biosynthetic pathway might be primed to produce specific very long chain fatty acids containing LacCer molecules, as required for the optimal physiological development of the neonate.

### 3.2. Quantification of GSLs Using LC-MS

The quantification of GSLs is complex because of the presence of multiple molecular species and the complex matrix in which they are measured. Different MS ionization responses caused by the different acyl fatty acid moieties, chain lengths and degrees of unsaturation also add to the complexity (Figure 7). Ideally, the quantification of Crb and LacCer should be carried out using standards that contain the same distribution of molecular species as those in the unknown samples, which was not practical for human milk. GSL standards purified from bovine milk were therefore the best option, as they provided a close match to the molecular distribution observed in human milk (Figure 5). Furthermore, despite the fact that human milk has been reported to contain predominantly GalCer [19], and the fact that the bovine-sourced Crb standard is GluCer dominated, we showed that both GluCer and GalCer (containing the same d-values) had the same ionization efficiencies, i.e., the polar head group had no significant impact on the ionization efficiency (Table S9).

Human milk was found to contain significantly higher concentrations of Crbs (9.9–17.4 μg/mL) than LacCer (1.3–3.0 μg/mL). It is known that Crbs are the precursors for the biosynthesis of other sphingolipids such as LacCer and gangliosides, as well as being an alternative metabolic source of sphingomyelin. It is therefore logical that higher reservoirs of Crb may be required to support rapid infant growth. Although a large variation in the concentrations of Crb and LacCer was observed between individual donors across lactation (Figure 8), the trends were similar; the Crb concentrations decreased gradually without any significant differences between time points, and there was no significant change in the LacCer concentrations. The relatively large fluctuations in the concentrations of LacCer between individuals was probably due to the accurate measurement of low concentrations that were close to the LOQ levels (1.6 µg/mL). This made it very challenging to accurately measure both LacCer and Crb using the same dilution of raw material. Interestingly, the GSL concentrations in the Malaysian cohort were generally lower than those measured for the Chinese cohort (Figure 8), although it is unclear if this was due to geographical or genetic differences, or was simply a consequence of the small sample size of this study.

In this study, three bovine milk samples of different batches were measured. The Crb concentration of 9.76–11.99 µg/mL (Table 4) in bovine milk was not significantly different from the average Crb concentration (9.9 ± 5.2 µg/mL) measured for the Malaysian cohort but was significantly less than that measured for the Chinese cohort (17.4 ± 7.0 µg/mL). Larger cohort studies are required to provide a better understanding of Crb concentration ranges, and to determine if there are indeed any differences based on the geographical location of the mother. Likewise, a better understanding of any seasonal changes in the Crb concentration in bovine milk is needed, given that most infant formulae are bovine milk based. This information is essential to improve the formulation of infant formula to make it a closer mimic of human breast milk. However, it is possible that this potential deficit could be mitigated by the significantly higher LacCer concentrations (14.25–16.16 μg/mL) found in bovine milk than in human milk (1.3–3.0 μg/mL, Table 4).

A comparison between MFGM Lipid 100 and bovine raw milk made on the basis of equivalent solids content showed that there were approximate four- and seven-fold increases in the concentrations of Crb and LacCer to 45 and 103 μg/mL, respectively. However, within this overall increase, the ratio of Crb to LacCer changed from approximately 1:1 in bovine milk to 1:2 in MFGM Lipid 100, possibly because of the different partitioning of Crb and LacCer during the manufacturing process. However, it should be noted that the raw materials used to produce MFGM Lipid 100 and the bovine milk tested in this study were not from the same origin in terms of species, time (season) and location, making this comparison purely indicative. Nevertheless, despite these differences, MFGM Lipid 100 could still be used to fortify Crb and LacCer in infant formula to produce a formulation that contains concentrations of these specific lipids that are deemed to be important for infant development, that more closely resemble those in human milk and that are typically depleted in current infant formula because of the use of vegetable oils instead of bovine milk fat.

## 4. Materials and Methods

### 4.1. Standards and Chemicals

Purified GluCer (from bovine buttermilk) and LacCer (from bovine buttermilk) were purchased from Matreya, LLC (Pleasant Gap, PA, USA). Synthesized pure GluCer standards d18:1/8:0, d18:1/12:0, d18:1/18:0, d18:1/18:1 and d18:1/24:1, GalCer standards d18:1/18:0, d18:1/18:0 (2S–OH) and d18:1/18:0 (2R–OH) and LacCer standards d18:1/8:0, d18:1/24:0 and d18:1/24:1 were purchased from Avanti Polar Lipids Inc. (Alabaster, AL, USA). All solvents used were purchased from Merck and were HPLC grade except for chloroform, which was analytical grade (stabilized with ethanol). Ammonium acetate was purchased from Fluka (Sigma Aldrich, St. Louis, MO, USA).

Standard stock solutions of 1 mg GluCer/mL and 1 mg LacCer/mL were prepared by adding 1 mL of chloroform/methanol (1:2, v/v) into vials that held 1 mg of each. Standard working solutions were diluted with acetonitrile/chloroform/methanol (3:1:2, v/v/v). All standard solutions were stored at –30 °C until use. Ammonium acetate was made to 500 mM with Milli-Q water as a stock solution. The water used in all experiments was filtered Milli-Q water.

### 4.2. Samples and Lipid Extraction

Three batches raw bovine milk samples, on different days from the factory silo, where each batch contains the pool milk from approximately 2000 individual cows, and a MFGM Lipid 100 were obtained from the Fonterra Research and Development Centre (Palmerston North, New Zealand). MFGM Lipid 100 is a commercial bovine-milk-enriched MFGM product of Fonterra Ltd., New Zealand. Chinese human milk samples of five donors from seven time points (0.5, 1, 2, 3, 4, 6 and 8 months) were obtained from the Guangzhou Women and Children’s Medical Centre, Guangzhou Hospital, China, with ethics approval permit 2014021201 [11]. Malaysian human milk samples of five donors from three time points (2, 6 and 12 months) were obtained from the Hospital University Sains Malaysia Pregnancy Cohort Study, with ethics approval permit NMRR-10-597-6110 [25].

The MFGM Lipid 100 sample was rehydrated in Milli-Q water to give a 2.5% *w/v* solution prior to extraction. All samples were extracted as described by Fong et al. [26] with minor modifications. Briefly, 0.5 mL of each sample was mixed with 2 mL of chloroform/methanol (1:2, *v/v*) and then well mixed by rocking for 20 min before centrifugation at 2000× *g* for 20 min. The supernatant was carefully transferred into a KIMAX tube and the pellet was rehydrated with 0.25 mL of water, before being re-extracted with 1 mL of chloroform/methanol (1:2, *v/v*). After another round of centrifugation at 2000× *g* for 20 min, the two supernatants were pooled, 0.65 mL of water was added and the mixture was briefly vortexed and then centrifuged at 2000× *g* for 30 min to partition the phases. The upper phase was discarded, and 0.25 mL of 0.01 M KCl and 0.375 mL of methanol were added to the lower phase. After vortexing, the mixture was again centrifuged at 2000× *g* for 30 min. The upper phase was discarded, and the lower phase containing the neutral lipids and the GSLs was transferred into a 5 mL volumetric flask, which was made up to the mark with acetonitrile/chloroform/methanol (3:1:2, *v/v/v*) before being subjected to HPLC-MS analysis. The extract from a randomly selected 8-month human milk sample from the Chinese cohort was used for the characterization work.

### 4.3. HPLC-MS Characterization of GSLs

The characterization study of each molecular specie in GluCer and LacCer standards was carried out by direct infusion of a mixture containing both standards (10 µg/mL each) at 5 µL/min into the mass spectrometer (TSQ Quantum Ultra EMR, Thermo Scientific, San Jose, CA, USA). The heated electrospray ionization (HESI) conditions were set as follows: spray voltage 3500 V, vaporizer temperature 50 °C, capillary temperature 240 °C, sheath and auxiliary gases 10 arbitrary units each, positive mode. Argon was used as collision gas. The collision energy (CE) used for MS^2^ and MS^3^ of both standards ranged from 18 to 35 arbitrary units.

Due to the major suppression of ionization associated with the sample matrix, which contains neutral lipids and phospholipids, the characterization study of GSLs (Crb and LacCer) in samples was firstly achieved by separation on a Luna HILIC column (250 mm x 4.6 mm, 5 µm, Phenomenex) using the an Agilent 1100 HPLC (Agilent, Santa Clara, CA, USA) system prior to MS analysis. Five microliters of sample from the autosampler (20 °C) was injected to the column, which was held at 30 °C. The HPLC mobile phase and gradient were adapted from Liu et al. [27]. The mobile phases consisted of acetonitrile with 0.1% formic acid (A) and 5 mM ammonium acetate (B). Analytes were eluted using a linear gradient from 5 to 21% B over the first 20 min, which was then decreased to 5% B over 1 min followed by being held at 5% B for another 4 min for column re-equilibration. The flow rate was set at 0.8 mL/min. The first 2 min containing neutral lipids was diverted to waste.

The HPLC system was interfaced to a mass spectrometer (LTQ-Orbitrap^TM^, Thermo Scientific, San Jose, CA, USA) with a HESI source and data collected in DDA mode. The ESI conditions were set as follows: spray voltage 5000 V, capillary temperature 320 °C, and sheath and auxiliary gases 30 and 54 arbitrary units, respectively. The full MS scan was collected in positive mode at a resolution of 30,000 with a mass range of *m/z* 650 to *m/z* 1100 and the MS^2^ settings used were dynamic exclusion time 45 s for the most intense ion with a collision-induced dissociation collision energy of 25 arbitrary units. Monoisotopic precursor ion selection was enabled.

GSL molecular species and fragments identification were manually made based on in-house database of possible species using literature fragmentation patterns.

### 4.4. HPLC-MS Quantification of GSLs

For quantitation the conditions were as described above (Section 4.3) but the HPLC used was different, namely, an Acquity Ultra Performance Liquid Chromatograph (UPLC, Waters, MA, USA). The analytes were ionized using an HESI source before being introduced into a triple quadrupole mass spectrometer (TSQ Quantum Ultra EMR, Thermo Scientific, San Jose, CA, USA) for the quantification analysis.

GluCer and LacCer standards were infused into the triple quadrupole mass spectrometer to optimize their fragmentation conditions. The optimized HESI settings were as follows: spray voltage 3000 V, vaporizer temperature 50 °C, sheath gas and auxiliary gas 10 arbitrary units each, capillary temperature 240 °C, positive mode. Argon was used as the collision gas with the CE for Crb and LacCer being set at 20 and 30 arbitrary units, respectively. GluCer and LacCer were identified by neutral losses of 180 and 342 amu, respectively in positive mode.

### 4.5. Validation–LOD, LOQ, Recovery and Reproducibility

The LOD and LOQ, defined as approximately three times the noise level for LOD and approximately 10 times the noise level for LOQ, were determined by injecting decreasing concentrations of the standards into the HPLC-MS/MS system.

In detail, GluCer (10 µg/mL) and LacCer (20 µg/mL) were spiked into both milk and MFGM Lipid 100 to assess their recovery rate in each sample matrix. Because of their different endogenous concentrations, the same concentrations of GluCer were spiked into human milk but lower concentrations of LacCer (5 µg/mL) were used.

The reproducibility of the method for Crb and LacCer quantification was evaluated using a human milk quality control sample, as well as a bovine milk sample and an MFGM Lipid 100 sample. These samples were measured in duplicate over 3 days.

A small shift in the retention time was observed with the HPLC HILIC column as it aged but was stable within each run. There were also small variations in the retention times observed between different HPLC Luna HILIC columns, but this did not have an impact on the quantification.

### 4.6. Impact of Polar Head Group and Fatty Acid Composition of GSL on MS Response

To assess the impact of the fatty acid composition on the MS response, each of the pure GluCer standards d18:1/8:0, d18:1/12:0, d18:1/18:0, d18:1/18:1 and d18:1/24:1 and the pure LacCer standards d18:1/8:0, d18:1/24:0 and d18:1/24:1 were reconstituted with acetonitrile/chloroform/methanol (3:1:2, *v/v/v*) to a concentration of 400 µM, which were then serially diluted to 200, 100, 50, 25, 12.5, 6.25, 3.125, 1.53 and 0.78 µM with acetonitrile/chloroform/methanol (3:1:2, *v/v/v*). Standard solutions were injected using a 5 µL injection loop to ensure that a fixed volume of the standard solution was introduced into the mass spectrometer.

To assess the impact of the type of polar head group on the MS response, GalCer d18:1/18:0 was reconstituted with acetonitrile/chloroform/methanol (3:1:2, *v/v/v*) to a concentration of 200 µM, which was then serially diluted to 100, 50, 25 and 12.5 µM, with acetonitrile/chloroform/methanol (3:1:2, *v/v/v*). These solutions were injected using the 5 µL injection loop from low to high concentrations and the results were compared with those from the GluCer d18:1/18:0 standard solutions of the same concentrations.

### 4.7. Statistical Analysis

All statistical analysis was conducted using Minitab (Release 16.2.4, 2013, Minitab Inc., State College, PA, USA). Comparison of the GSL results across different time points was conducted using a one-way analysis of variance, whereas comparison between two groups of data was conducted using paired t-tests.

## 5. Conclusions

This work reports the development of a robust method that identified and quantified 44 Crbs and 23 LacCers in human milk, bovine milk and MFGM Lipid 100 using HILIC chromatography coupled with MS*^n^* spectrometry.

The composition of human milk has always been the gold standard that manufacturers of infant formula aspire to match. Although a lot of emphasis has been placed on matching the concentrations of specific lipid components, it is also important to consider the differences in these components between different mammalian species. Human milk contains more GSLs with very long chain fatty acids, which are thought to be essential for infant development. Although bovine milk is widely used to make infant formulae, the distribution of the GSL types and their structures are different from those found in human milk. Therefore, the benefits of any bovine lipid supplement need to be clinically assessed [28,29]. Although this study has shown up differences in the distributions of complex GSLs between human and bovine milks, the sample numbers were small and changes over the lactation period of the cows were not investigated. Both these shortcomings need to be addressed before the differences in the distribution of various GSLs in human milk and bovine milk that were identified in this work can be confirmed. However, knowledge of these differences will enable the future development of processes that will offer selective enrichment of specific GSLs in MFGM-based ingredients to produce an infant formula that more nearly mimics human breast milk.

## Figures and Tables

**Table 1:** Proportion of distinct species containing specific long chain bases (LCB) in commercial standards, human milk ^a^, bovine milk and a commercial milk-fat-globule-membrane-enriched dairy ingredient (MFGM Lipid 100).

LCB	Cerebrosides (Crbs)				Lactosylceramide (LacCer)			
	Standard	Human Milk	Bovine Milk	MFGM Lipid 100	Standard	Human Milk	Bovine Milk	MFGM Lipid 100
15:0	0%	0%	4%	5%	0%	0%	3%	0%
15:1	0%	2%	0%	1%	0%	0%	3%	0%
16:0	11%	2%	3%	4%	18%	5%	13%	15%
16:1	7%	10%	23%	21%	18%	5%	13%	15%
17:0	7%	14%	6%	8%	0%	11%	11%	3%
17:1	0%	6%	13%	14%	6%	0%	11%	13%
18:0	18%	4%	1%	0%	24%	0%	8%	3%
18:1	36%	54%	39%	31%	35%	47%	29%	36%
18:2	0%	6%	0%	0%	0%	5%	0%	0%
19:0	7%	0%	0%	0%	0%	0%	0%	0%
19:1	7%	2%	6%	12%	0%	21%	5%	5%
20:0	4%	0%	0%	0%	0%	0%	0%	0%
20:1	4%	0%	1%	2%	0%	0%	3%	5%
21:4	0%	0%	0%	0%	0%	5%	3%	3%
22:1	0%	0%	0%	1%	0%	0%	0%	0%
22:2	0%	0%	0%	0%	0%	0%	0%	3%
23:1	0%	0%	4%	0%	0%	0%	0%	0%
24:1	0%	0%	0%	2%	0%	0%	0%	0%
Saturated	46%	20%	14%	17%	41%	16%	34%	21%
Monounsaturated	54%	74%	86%	83%	59%	74%	63%	74%
Polyunsaturated	0%	6%	0%	0%	0%	11%	3%	5%

^a^ The human milk results were based on a randomly selected eight-month sample from the Chinese cohort.

**Table 2:** Proportion of distinct species containing specific fatty acids in commercial standards, human milk ^a^, bovine milk and MFGM Lipid 100.

Fatty Amide	Cerebrosides (Crbs)				Lactosylceramide (LacCer)			
	Standard	Human Milk	Bovine Milk	MFGM Lipid 100	Standard	Human Milk	Bovine Milk	MFGM Lipid 100
13:2	0%	0%	0%	1%	0%	0%	0%	0%
14:0	0%	2%	1%	0%	0%	0%	0%	0%
14:2	0%	0%	0%	1%	0%	0%	0%	0%
15:0	0%	0%	0%	0%	0%	0%	0%	0%
15:1	0%	0%	1%	1%	0%	0%	0%	0%
15:2	0%	2%	1%	3%	0%	0%	0%	0%
16:0	4%	2%	7%	5%	12%	5%	5%	5%
16:1	4%	4%	3%	2%	0%	0%	0%	0%
16:2	0%	2%	1%	1%	0%	0%	0%	0%
16:3	0%	2%	1%	3%	0%	0%	0%	0%
16:0–OH	0%	2%	3%	3%	0%	0%	0%	0%
17:0	4%	2%	3%	1%	0%	5%	3%	3%
17:1	0%	2%	4%	3%	0%	0%	0%	0%
17:2	0%	6%	1%	3%	0%	0%	0%	0%
17:3	0%	4%	0%	0%	0%	0%	0%	0%
18:0	4%	6%	4%	4%	0%	11%	5%	8%
18:1	0%	4%	4%	4%	0%	0%	0%	0%
18:3	0%	0%	0%	1%	0%	0%	0%	0%
18:0–OH	0%	2%	6%	5%	0%	0%	0%	0%
19:0	0%	0%	0%	1%	0%	5%	0%	0%
19:1	0%	0%	4%	2%	0%	5%	0%	0%
19:2	0%	2%	0%	1%	0%	0%	0%	0%
20:0	0%	2%	0%	2%	0%	11%	3%	3%
20:1	0%	2%	0%	0%	0%	0%	0%	0%
20:2	0%	0%	1%	1%	0%	0%	3%	3%
19:1	0%	0%	0%	0%	0%	0%	0%	3%
21:0	4%	2%	1%	1%	0%	0%	3%	8%
21:2	0%	2%	4%	4%	0%	0%	5%	0%
22:0	14%	2%	3%	3%	24%	16%	18%	13%
22:1	4%	4%	3%	4%	0%	0%	0%	0%
22:2	0%	0%	6%	4%	0%	0%	8%	10%
22:4	0%	0%	0%	1%	0%	0%	0%	3%
22:0–OH	0%	4%	4%	3%	0%	0%	0%	0%
22:1–OH	0%	0%	0%	0%	0%	0%	0%	3%
23:0	32%	2%	7%	4%	29%	5%	11%	8%
23:1	4%	2%	3%	4%	0%	0%	8%	0%
23:2	0%	6%	4%	3%	0%	5%	5%	8%
23:3	0%	2%	0%	0%	0%	11%	3%	0%
23:0–OH	0%	2%	3%	4%	0%	0%	0%	0%
24:0	18%	6%	4%	4%	24%	11%	16%	13%
24:1	4%	4%	3%	3%	6%	0%	3%	0%
24:2	0%	0%	0%	1%	0%	0%	0%	0%
24:4	0%	2%	0%	0%	0%	0%	0%	0%
24:0–OH	0%	2%	4%	3%	0%	0%	0%	0%
25:0	7%	2%	0%	3%	6%	5%	3%	3%
25:1	0%	0%	0%	1%	0%	0%	0%	3%
25:2	0%	2%	1%	1%	0%	0%	0%	0%
25:0–OH	0%	4%	0%	0%	0%	0%	0%	0%
26:0	0%	2%	0%	2%	0%	0%	0%	3%
26:1	0%	0%	0%	2%	0%	0%	0%	0%
26:2	0%	0%	0%	0%	0%	0%	0%	3%
26:5	0%	0%	0%	0%	0%	5%	0%	3%
Saturated	86%	30%	31%	29%	94%	74%	66%	59%
Monounsaturated	14%	22%	26%	25%	6%	5%	11%	3%
Polyunsaturated	0%	32%	23%	28%	0%	21%	24%	36%
Saturated–OH	0%	16%	20%	17%	0%	0%	0%	0%
Unsaturated–OH	0%	0%	0%	0%	0%	0%	0%	3%

^a^ The human milk results were based on a randomly selected eight-month sample from the Chinese cohort.

**Table 3:** Summary of spike recovery rates.

	GluCer		LacCer		
Spiking concentrations	10 µg/mL	20 µg/mL	5 µg/mL	10 µg/mL	20 µg/mL
Human milk (*n* = 6)	96 ± 3.5%	106 ± 9.2%	109 ± 22.5%	115 ± 13.6%	
Bovine milk (*n* = 6)	111 ± 5.1%	101 ± 3.5%		96 ± 20.2%	95 ± 12.5%
MFGM Lipid 100 (*n* = 6)	99 ± 7.5%	108 ± 10.0%		97 ± 10.4%	103 ± 12.5%

**Table 4:** Average concentrations ± standard deviations of Crbs and LacCer in human milk, bovine milk and MFGM Lipid 100 (coefficients of variation in parentheses).

	Crbs (µg/mL)	LacCer (µg/mL)
Human milk (Chinese cohort) *	17.4 ± 7.0 ^a^	3.0 ± 2.0 ^a^
Human milk (Malaysian cohort) *	9.9 ± 5.2 ^b, c^	1.3 ± 0.9 ^b^
Human milk (*n* = 6)	9.10 ± 0.61 (6.7%) ^b, c^	1.35 ± 0.25 (18.7%) ^b^
Bovine milk 1 (*n* = 6)	11.99 ± 0.52 (4.3%) ^b, d^	14.74 ± 1.12 (7.6%) ^c^
Bovine milk 2 (*n* = 6)	11.61 ± 0.17 (1.5%) ^b, d^	16.16 ± 2.70 (16.7%) ^c^
Bovine milk 3 (*n* = 6)	9.76 ± 0.48 (5.0%) ^b, c^	14.25 ± 1.73 (12.2%) ^c^
MFGM Lipid 100 (*n* = 6) ^#^	447.9± 55.7 (12.4%)	1036.4 ± 115.4 (11.1%)

^a, b, c and d^ Results with the same superscript letter within the same class of glycosphingolipid show no significant difference (*p* > 0.05). * The averaged results in human milk from the Chinese and Malaysian cohorts were determined using all results measured across all time points (Figure 8). ^#^ Units are µg/g.

## Supplementary Materials

The following are available online, Table S1: List of GluCer molecular species found in the purified commercial standard with tentative identification based on accumulated MS, MS^2^ and MS^3^ data; Table S2: List of LacCer molecular species found in the purified commercial standard with tentative identification based on accumulated MS, MS^2^ and MS^3^ data; Table S3: List of Crb molecular species found in human milk with their tentative identification based on observed evidence; Table S4: List of LacCer molecular species found in human milk with their tentative identification based on observed evidence; Table S5: List of Crb molecular species found in bovine milk with their tentative identification based on observed evidence; Table S6: List of LacCer molecular species found in bovine milk with their tentative identification based on observed evidence; Table S7: List of Crb molecular species found in MFGM Lipid 100 with their tentative identification based on observed evidence; Table S8: List of LacCer molecular species found in MFGM Lipid 100 with their tentative identification based on observed evidence; Table S9: Responses of GalCer 18:1/18:0 and GluCer 18:1/18:0 at the same molar concentrations and their comparisons.
