# Supplementary material for: Qualitative and Quantitative Study of Glycosphingolipids in Human Milk and Bovine Milk Using High Performance Liquid Chromatography–Data-Dependent Acquisition–Mass Spectrometry
Source: Molecules. 2020 Sep 3;25(17):4024. doi: 10.3390/molecules25174024 (PMC7504816; doi:10.3390/molecules25174024)
Supplement: Supplementary file 1 [file molecules-25-04024-s001.pdf]

Supplementary Material

Qualitative and Quantitative Study of Glycosphingolipids in Human Milk and Bovine Milk Using High Performance Liquid Chromatography–Data-Dependent Acquisition–Mass Spectrometry

Lin Ma [1,*], Bertram Y. Fong [1], Alastair K. H. MacGibbon [1] and Gillian Norris [2]

[1] Fonterra Research and Development Centre, Dairy Farm Road, Private Bag 11029, Palmerston North 4442, New Zealand, Bertram.Fong@fonterra.com(B.Y.F.), alastair.macgibbon@fonterra.com(A.M.)

[2] Massey University, Private Bag 11222, Palmerston North 4442, New Zealand, G.Norris@massey.ac.nz

Table S1. List of GluCer molecular species found in the purified commercial standard with tentative identification based on accumulated MS, MS^2 and MS^3 data.

m/z	d-value	Backbone *m/z*	Sphingoid Base	Fatty Amide *m/z*	Fatty Acid	Tentative ID
698.6	d34:2	264.35	18:1 − H_2O	236.34	16:1	C18:1/16:1
700.6	d34:1	264.41	18:1 − H_2O	256.33	16:0	C18:1/16:0
716.6	d35:0	284.40	18:0	270.37	17:0	C18:0/17:0
728.6	d36:1	236.32	16:1 − H_2O			C16:1/20:0
		264.28	18:1 − H_2O	284.41	18:0	C18:1/18:0
744.6	d37:0	256.36	16:0 − H_2O	326.36	21:0	C16:0/21:0
756.6	d38:1	236.30	16:1 − H_2O	340.40	22:0	C16:1/22:0
		264.33	18:1 − H_2O			C18:1/20:0
770.6	d39:1	236.24	16:1 − H_2O	354.56	23:0	C16:1/23:0
		250.34	17:1 − H_2O			C17:1/22:0
		264.32	18:1 − H_2O			C18:1/21:0
772.6	d39:0	237.29	16:0 − H_2O	354.52	23:0	C16:0/23:0
		251.20	17:0 − H_2O	340.31	22:0	C17:0/22:0
782.6	d40:2	264.31	18:1 − H_2O	355.54	22:1	C18:1/22:1
784.6	d40:1	236.29	16:1 − H_2O			C16:1/24:0
		250.36	17:1 − H_2O			C17:1/23:0
		264.32	18:1 − H_2O	340.45	22:0	C18:1/22:0
786.6	d40:0	237.44	16:0 − H_2O	340.54	22:0	C16:0/24:0
		252.53	17:0 − H_2O	354.52	23:0	C17:0/23:0
		266.34	18:0 − H_2O	368.48	24:0	C18:0/22:0
796.6	d41:2	250.25	17:1 − H_2O			C17:1/24:1
		264.31	18:1 − H_2O	352.34	23:1	C18:1/23:1
798.6	d41:1	250.27	17:1 − H_2O			C17:1/24:0
		264.29	18:1 − H_2O	354.44	23:0	C18:1/23:0
800.6	d41:0	266.43	18:0 − H_2O	354.53	23:0	C18:0/23:0
810.6	d42:2	250.34	17:1 − H_2O			C17:1/25:1
		264.30	18:1 − H_2O	366.28	24:1	C18:1/24:1
		278.35	19:1 − H_2O			C19:1/23:1

812.6	d42:1	264.34	18:1 – H_2O	368.55	24:0	C18:1/24:0
		278.33	19:1 – H_2O	354.41	23:0	C19:1/23:0
814.6	d42:0	266.38	18:0 – H_2O	368.52	24:0	C18:0/24:0
		280.30	19:0 – H_2O	354.40	23:0	C19:0/23:0
826.6	d43:1	264.27	18:1 – H_2O	382.62	25:0	C18:1/25:0
		278.29	19:1 – H_2O	368.51	24:0	C19:1/24:0
		292.34	20:1 – H_2O	354.48	23:0	C20:1/23:0
828.6	d43:0	267.51	18:1 – H_2O	382.50	25:0	C18:0/25:0
		280.49	19:1 – H_2O	368.56	24:0	C19:0/24:0
		294.51	20:0 – H_2O	354.54	23:0	C20:0/23:0

Table S2. List of LacCer molecular species found in the purified commercial standard with tentative identification based on accumulated MS, MS^2 and MS^3 data.

m/z	d-value	Backbone *m/z*	Sphingoid Base	Fatty Amide *m/z*	Fatty Acid	Tentative ID
862.6	d34:1	264.36	18:1 – H_2O	256.44	16:0	C18:1/16:0
864.6	d34:0	266.32	18:0 – H_2O	256.36	16:0	C18:0/16:0
918.6	d38:1	236.25	16:1 – H_2O	340.58	22:0	C16:1/22:0
		264.38	18:1 – H_2O			C18:1/20:0
920.6	d38:0	256.37	16:0	340.41	22:0	C16:0/22:0
932.6	d39:1	236.23	16:1	354.49	23:0	C16:1/23:0
		250.34	17:1			C17:1/22:0
		264.29	18:1			C18:1/21:0
934.6	d39:0	256.36	16:0	354.51	23:0	C16:0/23:0
946.6	d40:1	236.26	16:1	340.52	22:0	C16:1/24:0
		250.39	17:1	354.36	23:0	C17:1/23:0
		264.29	18:1	368.45	24:0	C18:1/22:0
948.6	d40:0	256.38	16:0	340.41	22:0	C16:0/24:0
		284.42	18:0	368.49	24:0	C18:0/22:0
960.6	d41:1	250.32	17:1	354.48	23:0	C17:1/24:0
		264.34	18:1			C18:1/23:0
		278.38	19:1			C19:1/22:0
962.6	d41:0	284.35	18:0	354.46	23:0	C18:0/23:0
		298.63	19:0			C19:0/22:0
972.6	d42:2	278.30	16:1			C16:1/26:1
		264.97	18:1	366.17	24:1	C18:1/24:1
974.6	d42:1	264.31	18:1	368.35	24:0	C18:1/24:0
976.6	d42:0	266.22	18:0	368.53	24:0	C18:0/24:0
988.6	d43:1	264.34	18:1	382.19	25:0	C18:1/25:0

Table S3. List of Crb molecular species found in human milk with their tentative identification based on observed evidence.

m/z	d-value	Backbone *m/z*	Sphingoid Base	Fatty Amide *m/z*	Fatty Acid	Tentative ID
654.36	d31:3	264.27	18:1 – H_2O			C18:1/13:2
670.56	d32:2	236.24	16:1 – H_2O	236.24	16:1 – H_2O	C16:1/16:1

		262.26	18:2 − H_2O			C18:2/14:0
		264.27	18:1 − H_2O			C18:1/14:1
672.38	d32:1	264.27	18:1 − H_2O	228.13	14:0	C18:1/14:0
674.48	d32:0 or d31:1–OH	228.13	14:0			C14:0/18:0
		264.27	18:1 − H_2O			C18:1/13:0–OH
682.39	d33:3	236.24	16:1 − H_2O	266.28	17:2	C16:1/17:2
		252.27	17:0 − H_2O	250.25	16:3	C17:0/16:3
		256.26	16:0	264.27	17:3	C16:0/17:3
		264.27	18:1 − H_2O	256.26	15:2 + H_2O	C18:1/15:2
698.56	d34:2	252.36	17:0 − H_2O	266.51	17:2	C17:0/17:2
		264.38	18:1 − H_2O	236.33	16:1 − H_2O	C18:1/16:1
		266.51	18:0 − H_2O	252.36	16:2	C18:0/16:2
700.53	d34:1	254.30	16:1	266.40	18:0 − H_2O	C16:1/18:0
		264.49	18:1	256.36	16:0 − H_2O	C18:1/16:0
702.50	d33:1–OH	264.27	18:1 − H_2O			C18:1/15:0–OH
710.59	d35:3	236.24	16:1 − H_2O			C16:1/19:2
		252.27	17:0 − H_2O			C17:0/18:3
		262.25	18:2 − H_2O	250.25	17:1 − H_2O	C18:2/17:1
		264.27	18:1 − H_2O	266.28	17:2	C18:1/17:2
		284.30	18:0	264.27	17:3	C18:0/17:3
712.57	d35:2	250.25	17:1 − H_2O	264.27	18:1 − H_2O	C17:1/18:1
		262.25	18:2 − H_2O			C18:2/17:0
714.59	d35:1	250.25	17:1 − H_2O	284.29	18:0	C17:1/18:0
		264.27	18:1 − H_2O	252.27	17:0 − H_2O	C18:1/17:0
716.57	d34:1–OH	264.49	18:1 − H_2O	272.00	16:0 − H + OH	C18:1/16:0–OH
				300.25	18:0 − H + OH	C16:1/18:0–OH
726.62	d36:2	252.27	17:0 − H_2O			C17:0/19:2
		264.27	18:1 − H_2O	264.27	18:1 − H_2O	C18:1/18:1
728.60	d36:1	264.33	18:1 − H_2O	284.30	18:0	C18:1/18:0
736.39	d37:4	236.24	16:1 − H_2O			C16:1/21:3
		250.25	17:1 − H_2O			C17:1/20:3
		252.27	17:0 − H_2O			C17:0/20:4
		262.25	18:2 − H_2O			C18:2/19:2
		264.27	18:1 − H_2O			C18:1/19:3
738.65	d37:3	236.24	16:1 − H_2O			C16:1/21:2
		252.27	17:0 − H_2O			C17:0/20:3
		262.25	18:2 − H_2O			C18:2/19:1
		264.27	18:1 − H_2O	312.33	19:2 + H_2O	C18:1/19:2
740.64	d37:2	264.27	18:1 − H_2O			C18:1/19:1
774.40	d36:1–OH	264.27	18:1 − H_2O	300.29	18:0 − H + OH	C18:1/18:0–OH
752.63	d38:3	250.25	17:1 − H_2O			C17:1/21:2
		262.25	18:2 − H_2O			C18:2/20:1
		264.27	18:1 − H_2O			C18:1/20:2
754.65	d38:2	224.24	15:0 − H_2O			C15:0/23:2
		236.24	16:1 − H_2O	338.34	22:1	C16:1/22:1

		252.27	17:0 − H_2O			C17:0/21:2
		262.25	18:2 − H_2O			C18:2/20:0
		264.27	18:1 − H_2O	310.31	20:1	C18:1/20:1
		310.31	20:1			C20:1/18:0
756.63	d38:1	236.36	16:1 − H_2O	312.63	20:0	C16:1/22:0
		264.40	18:1 − H_2O			C18:1/20:0
		280.29	19:0 − H_2O			C19:0/19:1
766.53	d39:3	236.24	16:1 − H_2O			C16:1/23:2
		250.25	17:1 − H_2O			C17:1/22:2
		252.27	17:0 − H_2O			C17:0/22:3
		262.25	18:2 − H_2O			C18:2/21:1
		264.27	18:1 − H_2O	340.36	21:2 + H_2O	C18:1/21:2
768.55	d39:2	252.27	17:0 − H_2O			C17:0/22:2
		262.25	18:2 − H_2O			C18:2/21:0
		264.27	18:1 − H_2O			C18:1/21:1
770.57	d39:1	250.48	17:1 − H_2O	326.79	21:0	C17:1/22:0
		264.34	18:1 − H_2O			C18:1/21:0
		278.36	19:1 − H_2O			C19:1/20:0
772.58	d38:1−OH	236.19	16:1			C16:1/22:0
		264.36	18:1			C18:1/20:0
780.67	d40:3	250.25	17:1 − H_2O			C17:1/23:2
		252.27	17:0 − H_2O			C17:0/23:3
		262.25	18:2 − H_2O			C18:2/22:1
		264.27	18:1 − H_2O			C18:1/22:2
782.65	d40:2	224.47	15:0 − H_2O			C15:0/25:2
		236.37	16:1 − H_2O	366.84	24:1	C16:1/24:1
		252.42	17:0 − H_2O	368.63	23:2	C17:0/23:2
		262.46	18:2 − H_2O			C18:2/22:0
		264.41	18:1 − H_2O	338.74	22:1	C18:1/22:1
784.67	d40:1	250.18	17:1 − H_2O	340.63	22:0	C17:1/23:0
		264.26	18:1 − H_2O			C18:1/22:0
		280.39	19:0 − H_2O			C19:0/21:1
786.60	d40:0 or d39:1−OH	236.24	16:1 − H_2O	356.35	22:0−H + OH	C16:1/23:0−OH
		250.25	17:1 − H_2O			C17:1/22:0−OH
		252.27	17:0 − H_2O			C17:0/23:0
		264.27	18:1 − H_2O			C18:1/21:0−OH
792.55	d41:4	250.25	17:1 − H_2O			C17:1/24:3
		252.27	17:0 − H_2O	378.37	24:4 + H_2O	C17:0/24:4
		262.25	18:2 − H_2O	368.39	23:2 + H_2O	C18:2/23:2
		264.27	18:1 − H_2O	366.37	23:3 + H_2O	C18:1/23:3
794.60	d41:3	250.25	17:1 − H_2O			C17:1/24:2
		252.27	17:0 − H_2O			C17:0/24:3
		262.25	18:2 − H_2O			C18:1/23:1
		264.27	18:1 − H_2O	368.39	23:2 + H_2O	C18:1/23:2
796.62	d41:2	250.14	17:1 − H_2O			C17:1/24:1
		264.42	18:1 − H_2O	370.27	23:1 + H_2O	C18:1/23:1

		278.63	19:1 − H_2O			C19:1/22:1
798.62	d41:1	252.21	17:0 − H_2O	354.64	23:0	C17:0/24:1
		264.37	18:1 − H_2O			C18:1/23:0
		278.38	19:1 − H_2O			C19:1/22:0
800.57	d41:0 or d40:1–OH	224.24	15:0 − H_2O			C15:0/26:0
		236.24	16:1 − H_2O			C16:1/24:0–OH
		240.24	15:1	398.36	25:0 − H + OH	C15:1/25:0–OH
		252.27	17:0 − H_2O			C17:0/24:0
		264.27	18:1 − H_2O	356.35	22:0 − H + OH	C18:1/22:0–OH
810.69	d42:2	252.24	17:0 − H_2O	396.64	25:2 + H_2O	C17:0/25:2
		262.18	18:2 − H_2O	368.62	24:0	C18:2/24:0
		264.37	18:1 − H_2O	366.55	24:1	C18:1/24:1
		278.51	19:1 − H_2O			C19:1/23:1
812.62	d42:1	252.31	17:0 − H_2O	368.69	24:0	C17:0/25:1
		264.39	18:1 − H_2O			C18:1/24:0
		298.59	19:0			C19:0/23:1
814.63	d41:1–OH	250.32	17:1 − H_2O			C17:1/24:0–OH
		264.45	18:1 − H_2O	352.38	23:0 − H + OH	C18:1/23:0–OH
824.70	d43:2	250.25	17:1 − H_2O			C17:1/26:1
		252.27	17:0 − H_2O			C17:0/26:2
		262.25	18:2 − H_2O			C18:2/25:0
		264.27	18:1 − H_2O			C18:1/25:1
826.72	d43:1	250.33	17:1 − H_2O			C17:1/26:0
		252.49	17:0 − H_2O			C17:0/26:1
		264.63	18:1 − H_2O	382.03	25:0	C18:1/25:0
		278.25	19:1 − H_2O	368.54	24:0	C19:1/24:0
828.69	d43:0 or d42:1–OH	252.27	17:0 − H_2O	396.38	25:1 − H + OH	C17:0/25:0–OH
		252.27	17:0 − H_2O	396.38	26:0	C17:0/26:0
		264.27	18:1 − H_2O	384.38	24:0 − H + OH	C18:1/24:0–OH
838.63	d44:2	240.24	15:1			C15:1/29:1
		262.26	18:2 − H_2O			C18:2/26:0
		264.27	18:1 − H_2O			C18:1/26:1
		290.28	20:2 − H_2O			C20:2/24:0
		292.30	20:1 − H_2O			C20:1/24:1

Table S4. List of LacCer molecular species found in human milk with their tentative identification based on observed evidence.

m/z	d-value	Backbone *m/z*	Sphingoid Base	Fatty Amide *m/z*	Fatty Acid	Tentative ID
862.63	d34:1	264.42	18:1 − H_2O	256.48	16:0	C18:1/16:0
864.70	d34:0 or d33:1–OH	236.24	16:1 − H_2O			C16:1/17:0–OH
		252.27	17:0 − H_2O	252.27	17:0 − H_2O	C17:0/C17:0
		256.26	16:0	284.29	18:0	C16:0/18:0
		264.27	18:1 − H_2O			C18:1/15:0–OH
890.65	d36:1	224.24	15:0 − H_2O			C15:0/21:1
		236.24	16:1 − H_2O			C16:1/20:0

		252.27	17:0 − H_2O	314.28	19:1 + H_2O	C17:0/19:1
		264.27	18:1 − H_2O	284.29	18:0	C18:1/18:0
914.78	d38:3	264.27	18:1 − H_2O			C18:1/20:2
916.78	d38:2	264.27	18:1 − H_2O			C18:1/20:1
		280.27	19:0 − H_2O			C19:0/19:2
918.69	d38:1	236.35	16:1 − H_2O	340.72	22:0	C16:1/22:0
		264.41	18:1 − H_2O	312.47	20:0	C18:1/20:0
		278.05	19:1 − H_2O	316.34	19:0 + H_2O	C19:1/19:0
930.84	d39:2	236.24	16:1 − H_2O			C16:1/23:1
		252.27	17:0 − H_2O			C17:0/22:2
		264.27	18:1 − H_2O			C18:1/21:1
932.71	d39:1	278.27	19:1 − H_2O	330.21	20:0 + H_2O	C19:1/20:0
946.72	d40:1	236.48	16:1 − H_2O			C16:1/24:0
		264.42	18:1 − H_2O	340.67	22:0	C18:1/22:0
		288.30	17:0 + H_2O			C17:0/23:1
954.72	d41:4	252.27	17:0 − H_2O			C17:0/24:4
		262.25	18:2 − H_2O	368.39	23:2 + H_2O	C18:2/23:2
		264.27	18:1 − H_2O	366.37	23:3 + H_2O	C18:1/23:3
956.65	d41:3	234.12	16:2 − H_2O			C16:2/25:1
		264.27	18:1 − H_2O			C18:1/23:2
960.74	d41:1	264.35	18:1 − H_2O	354.28	23:0	C18:1/23:0
		278.39	19:1 − H_2O	358.26	22:0 + H_2O	C19:1/22:0
974.75	d42:1	252.27	17:0 − H_2O			C17:0/25:1
		264.27	18:1 − H_2O	368.39	24:0	C18:1/24:0
988.77	d43:1	264.42	18:1 − H_2O	400.27	25:0 + H_2O	C18:1/25:0
		278.29	19:1 − H_2O	386.08	24:0 + H_2O	C19:1/24:0
990.74	d43:0 or d42:1–OH	264.27	18:1 − H_2O			C18:1/24:0–OH
				300.29	18:0 − H + OH	C24:1/18:0–OH
992.76	d44:6	264.27	18:1 − H_2O	386.07	26:5	C18:1/26:5
		300.29	21:4 − H_2O	368.39	23:2 + H_2O	C21:4/23:2
		318.30	22:2 − H_2O			C22:2/22:4

Table S5. List of Crb molecular species found in bovine milk with their tentative identification based on observed evidence.

m/z	d-value	Backbone *m/z*	Sphingoid Base	Fatty Amide *m/z*	Fatty Acid	Tentative ID
670.56	d32:2	236.24	16:1 − H_2O	236.24	16:1 − H_2O	C16:1/16:1
		250.25	17:1 − H_2O			C17:1/15:1
		264.27	18:1 − H_2O			C18:1/14:1
672.37	d32:1	224.24	15:0 − H_2O	250.25	17:1 − H_2O	C15:0/17:1
		236.24	16:1 − H_2O	256.26	16:0	C16:1/16:0
		252.27	17:0 − H_2O			C17:0/15:1
		264.27	18:1 − H_2O	228.13	14:0	C18:1/14:0
674.48	d32:0 or d31:1–OH	236.24	16:1 − H_2O			C16:1/15:0–OH
		264.27	18:1 − H_2O			C18:1/13:0–OH

682.60	d33:3	252.27	17:0 − H_2O	268.26	16:3 + H_2O	C17:0/16:3
		264.27	18:1 − H_2O	256.26	15:2 + H_2O	C18:1/15:2
684.61	d33:2	250.25	17:1 − H_2O			C17:1/16:1
		252.27	17:0 − H_2O	252.27	16:2	C17:0/16:2
		264.27	18:1 − H_2O	240.10	15:1	C18:1/15:1
686.51	d33:1	250.25	17:1 − H_2O	256.26	16:0	C17:1/16:0
		264.27	18:1 − H_2O			C18:1/15:0
698.56	d34:2	264.58	18:1 − H_2O	272.48	16:1 + H_2O	C18:1/16:1
700.49	d34:1	252.27	17:0 − H_2O	286.01	17:1 + H_2O	C17:0/17:1
		264.27	18:1 − H_2O	256.26	16:0	C18:1/16:0
702.51	d34:0 or d33:1–OH	256.26	16:0	284.29	18:0	C16:0/18:0
		252.27	17:0 − H_2O	252.27	17:0 − H_2O	C17:0/17:0
		284.29	18:0	256.26	16:0	C18:0/16:0
		264.27	18:1 − H_2O			C18:1/15:0–OH
710.59	d35:3	236.24	16:1 − H_2O			C16:1/19:2
		252.27	17:0 − H_2O			C17:0/18:3
		264.27	18:1 − H_2O	284.29	17:2 + H_2O	C18:1/17:2
712.61	d35:2	236.24	16:1 − H_2O	278.28	19:1 − H_2O	C16:1/19:1
		250.25	17:1 − H_2O	264.27	18:1 − H_2O	C17:1/18:1
		264.27	18:1 − H_2O	250.25	17:1 − H_2O	C18:1/17:1
714.51	d35:1	238.25	16:0 − H_2O	296.29	19:1	C16:0/19:1
		250.25	17:1 − H_2O	266.28	18:0 − H_2O	C17:1/18:0
		264.27	18:1 − H_2O	252.27	17:0 − H_2O	C18:1/17:0
		278.28	19:1 − H_2O	256.26	16:0	C19:1/16:0
716.40	d35:0 or d34:1–OH	236.24	16:1 − H_2O	300.29	18:0 − H + OH	C16:1/18:0–OH
		252.27	17:0 − H_2O			C17:0/18:0
		264.27	18:1 − H_2O	272.26	16:0 − H + OH	C18:1/16:0–OH
726.62	d36:2	236.24	16:1 − H_2O			C16:1/20:1
		252.27	17:0 − H_2O			C17:0/19:2
		264.27	18:1 − H_2O	282.28	18:1	C18:1/18:1
728.61	36:1	252.68	17:0 − H_2O			C17:0/19:1
		264.45	18:1 − H_2O	284.51	18:0	C18:1/18:0
738.66	d37:3	224.24	15:0 − H_2O			C15:0/22:3
		236.24	16:1 − H_2O	340.36	21:2 + H_2O	C16:1/21:2
		264.27	18:1 − H_2O			C18:1/19:2
740.52	d37:2	224.24	15:0 − H_2O	336.76	22:2	C15:0/22:2
		236.24	16:1 − H_2O			C16:1/21:1
		250.25	17:1 − H_2O			C17:1/20:1
		264.27	18:1 − H_2O	278.28	19:1 − H_2O	C18:1/19:1
		278.28	19:1 − H_2O	264.27	18:1 − H_2O	C19:1/18:1
744.55	d37:0 or d36:1–OH	252.27	17:0 − H_2O			C17:0/20:0
		264.27	18:1 − H_2O	300.29	18:0 − H + OH	C18:1/18:0–OH
752.64	d38:3	224.24	15:0 − H_2O			C15:0/23:3
		236.24	16:1 − H_2O	354.37	22:2 + H_2O	C16:1/22:2
		250.25	17:1 − H_2O	340.36	21:2 + H_2O	C17:1/21:2

		264.27	18:1 − H_2O	326.34	20:2 + H_2O	C18:1/20:2
754.65	d38:2	224.24	15:0 − H_2O			C15:0/23:2
		236.24	16:1 − H_2O	356.35	22:1 + H_2O	C16:1/22:1
756.64	d38:1	236.24	16:1 − H_2O	340.53	22:0	C16:1/22:0
		250.47	17:1 − H_2O			C17:1/21:0
		264.39	18:1 − H_2O			C18:1/20:0
766.53	d39:3	236.24	16:1 − H_2O	368.39	23:2 + H_2O	C16:1/23:2
		250.25	17:1 − H_2O			C17:0/22:3
		252.27	17:0 − H_2O	354.37	22:2 + H_2O	C17:1/22:2
		264.27	18:1 − H_2O	340.36	21:2 + H_2O	C18:1/21:2
768.55	d39:2	236.24	16:1 − H_2O	370.37	23:1 + H_2O	C16:1/23:1
		250.25	17:1 − H_2O			C17:1/22:1
		264.27	18:1 − H_2O			C18:1/21:1
770.65	d39:1	236.27	16:1 − H_2O	354.54	23:0	C16:1/23:0
		250.27	17:1 − H_2O			C17:1/22:0
		252.37	17:0 − H_2O			C17:0/22:1
		264.33	18:1 − H_2O	326.60	21:0	C18:1/21:0
772.27	d38:1–OH	236.24	16:1 − H_2O	356.07	22:0 − H + OH	C16:1/22:0–OH
		250.25	17:1 − H_2O			C17:1/21:0–OH
		264.27	18:1 − H_2O			C18:1/20:0–OH
780.55	d40:3	236.24	16:1 − H_2O			C16:1/24:2
		252.27	17:1 − H_2O	368.39	23:2 + H_2O	C17:1/23:2
		264.27	18:1 − H_2O	354.37	22:2 + H_2O	C18:1/22:2
782.57	d40:2	224.24	15:0 − H_2O	396.38	25:2 + H_2O	C15:0/25:2
		236.24	16:1 − H_2O	384.38	24:1 + H_2O	C16:1/24:1
		250.25	17:1 − H_2O			C17:1/23:1
		264.27	18:1 − H_2O	356.35	22:1 + H_2O	C18:1/22:1
784.66	d40:1	236.24	16:1 − H_2O	368.29	24:0	C16:1/24:0
		250.25	17:1 − H_2O	354.37	23:0	C17:1/23:0
		252.27	17:0 − H_2O			C17:0/23:1
		264.27	18:1 − H_2O	340.45	22:0	C18:1/22:0
786.53	d40:0 or d39:1–OH	224.24	15:0 − H_2O			C15:0/25:0
		236.24	16:1 − H_2O	370.37	23:0 − H + OH	C16:1/23:0–OH
		250.25	17:1 − H_2O	356.35	22:0 − H + OH	C17:1/22:0–OH
		254.25	18:1 − H_2O			C18:1/21:0–OH
		352.36	23:1	272.26	16:0 − H + OH	C23:1/16:0–OH
794.57	d41:3	264.27	18:1 − H_2O	368.39	23:2 − H_2O	C18:1/23:2
796.67	d41:2	236.38	16:1 − H_2O			C16:1/25:1
		250.45	17:1 − H_2O			C17:1/24:1
		264.42	18:1 − H_2O	370.73	23:1 + H_2O	C18:1/23:1
798.69	d41:1	236.22	16:1 − H_2O			C16:1/25:0
		250.09	17:1 − H_2O			C17:1/24:0
		264.43	18:1 − H_2O	354.60	23:0	C18:1/23:0
		278.53	19:1 − H_2O			C19:1/22:0
800.58	d41:0 or d40:1–OH	236.24	16:1 − H_2O	384.38	24:0 − H + OH	C16:1/24:0–OH
		250.25	17:1 − H_2O			C17:1/23:0–OH

		264.27	18:1 − H_2O	356.35	23:0 − H + OH	C18:1/22:0–OH
		252.27	17:0 − H_2O			C17:0/25:2
810.75	d42:2	264.27	18:1 − H_2O	366.37	24:1	C18:1/24:1
		278.28	19:1 − H_2O			C19:1/23:1
		264.34	18:1 − H_2O	368.29	24:0	C18:1/24:0
812.62	d42:1	278.55	19:1 − H_2O	354.68	23:0	C19:1/23:0
		292.11	20:1 − H_2O			
		236.24	16:1 − H_2O			C16:1/25:0–OH
		238.25	16:0 − H_2O			C16:0/26:0
		250.25	17:1 − H_2O	384.38	24:0 − H + OH	C17:1/24:0–OH
814.56	d42:0 or d41:1–OH	252.27	17:0 − H_2O			C17:0/25:0
		264.27	18:1 − H_2O	370.37	23:0 − H + OH	C18:1/23:0–OH
		278.28	19:1 − H_2O			C19:1/22:0–OH
		352.36	23:1	300.29	18:0 − H + OH	C23:1/18:0–OH
		264.34	18:1 − H_2O			C18:1/25:0
826.71	d43:1	278.34	19:1 − H_2O	350.02	24:0 − H_2O	C19:1/24:0
		292.02	20:1 − H_2O	354.36	23:0	C20:1/23:0
		236.24	16:1 − H_2O			C16:1/26:0–OH
		252.27	17:0 − H_2O			C17:0/26:0
828.69	d43:0 or d42:1–OH	264.27	18:1 − H_2O	384.38	24:0 − H + OH	C18:1/24:0–OH
		278.28	19:1 − H_2O			C19:1/23:0–OH
		366.37	24:1 − H_2O	300.29	18:0 − H + OH	C23:1/18:0–OH

Table S6. List of LacCer molecular species found in bovine milk with their tentative identification based on observed evidence.

m/z	d-value	Backbone *m/z*	Sphingoid Base	Fatty Amide *m/z*	Fatty Acid	Tentative ID
862.63	d34:1	236.39	16:1 − H_2O			C16:1/18:0
		264.31	18:1 − H_2O	256.43	16:0	C18:1/16:0
		252.27	17:0 − H_2O	252.27	17:0	C17:0/17:0
864.76	d34:0 or d33:1–OH	256.26	16:0	284.27	18:0	C16:0/18:0
		264.27	18:1 − H_2O			C18:1/15:0–OH
		284.29	18:0	256.26	16:0	C18:0/16:0
		236.24	16:1 − H_2O			C16:1/20:0
890.65	d36:1	256.26	16:0			C16:0/20:1
		264.27	18:1 − H_2O	284.29	18:0	C18:1/18:0
		224.24	15:0 − H_2O			C15:0/23:3
914.69	d38:3	236.24	16:1 − H_2O	354.37	22:2 + H_2O	C16:1/22:2
		250.25	17:1 − H_2O	340.36	21:2 + H_2O	C17:1/21:2
		264.27	18:1 − H_2O	290.28	20:2 − H_2O	C18:1/20:2
		224.24	15:0 − H_2O	352.95	23:1	C15:1/23:1
918.69	d38:1	236.24	16:1 − H_2O	340.36	22:0	C16:1/22:0
		264.27	18:1 − H_2O	358.70	22:0 + H_2O	C18:1/20:0
		274.27	16:0 + H_2O			C16:0/22:1
920.82	d38:0 or d37:1–OH	236.24	16:1 − H_2O			C16:1/21:0–OH
		256.26	16:0	340.36	22:0	C16:0/22:0

928.71	d39:3	224.24	15:0 − H_2O			C15:0/24:3
		236.24	16:1 − H_2O	368.39	23:2 + H_2O	C16:1/23:2
		250.25	17:1 − H_2O	354.37	22:2 + H_2O	C17:1/22:2
		264.27	18:1 − H_2O	340.36	21:2 + H_2O	C18:1/21:2
932.70	d39:1	224.24	15:0 − H_2O			C15:0/24:1
		236.24	16:1 − H_2O	354.37	23:0	C16:1/23:0
		250.25	17:1 − H_2O	340.36	22:0	C17:1/22:0
		256.26	16:0	352.36	23:1	C16:0/23:1
		264.27	18:1 − H_2O	326.34	21:0	C18:1/21:0
934.72	d39:0 or d38:1–OH	224.24	15:0 − H_2O	386.12	24:0 + H_2O	C15:0/24:0
		236.24	16:1 − H_2O			C16:1/22:0–OH
		250.25	17:1 − H_2O			C17:1/21:0–OH
		256.26	16:0	354.37	23:0	C16:0/23:0
		270.28	17:0	340.36	22:0	C17:0/22:0
		284.29	18:0			C18:0/21:0
942.64	d40:3	236.24	16:1 − H_2O			C16:1/24:2
		250.25	17:1 − H_2O			C17:1/23:2
		252.27	17:0 − H_2O	366.37	23:3 + H_2O	C17:0/23:3
		264.27	18:1 − H_2O	354.37	22:2 + H_2O	C18:1/22:2
946.72	d40:1	236.37	16:1 − H_2O	368.62	C24:0	C16:1/24:0
		250.14	17:1 − H_2O			C17:1/23:0
		252.39	17:0 − H_2O	370.46	23:1 − H_2O	C17:0/23:1
		264.26	18:1 − H_2O	340.49	22:0	C18:1/22:0
948.73	d40:0 or d39:1–OH	236.24	16:1 − H_2O			C16:1/23:0–OH
		256.26	16:0	368.39	24:0	C16:0/24:0
		264.27	18:1 − H_2O			C18:1/21:0–OH
		284.29	18:0	340.36	22:0	C18:0/22:0
960.74	d41:1	236.47	16:1 − H_2O			C16:1/25:0
		250.27	17:1 − H_2O	386.12	24:0 + H_2O	C17:1/24:0
		252.27	17:0 − H_2O			C17:0/24:1
		264.27	18:1 − H_2O	354.37	23:0	C18:1/23:0
		278.28	19:1 − H_2O			C19:1/22:0
974.75	d42:1	236.24	16:1 − H_2O			C16:1/26:0
		252.27	17:0 − H_2O			C17:1/25:0
		264.27	18:1 − H_2O	368.39	24:0	C18:1/24:0
		278.28	19:1 − H_2O	354.37	23:0	C19:1/23:0
		284.29	18:0	366.37	24:1	C18:0/24:1
		292.30	20:1 − H_2O	340.36	22:0	C20:1/22:0
988.77	d43:1	252.27	17:0 − H_2O			C17:0/26:1
		264.27	18:1 − H_2O	382.40	25:0	C18:1/25:0
		278.28	19:1 − H_2O	368.39	24:0	C19:1/24:0
		292.30	20:1 − H_2O			C20:1/23:0
992.76	d44:6	264.27	18:1 − H_2O			C18:1/26:5
		300.29	21:4 − H_2O	368.39	23:2 + H_2O	C21:4/23:2
		318.30	22:2 − H_2O			C22:2/22:4
1002.78	d44:1	252.27	17:0 − H_2O			C17:0/27:1

		264.27	18:1 – H_2O			C18:1/26:0
		278.28	19:1 – H_2O			C19:1/25:0
		292.30	20:1 – H_2O			C20:1/24:0

Table S7. List of Crb molecular species found in MFGM Lipid 100 with their tentative identification based on observed evidence.

m/z	d-value	Backbone *m/z*	Sphingoid Base	Fatty Amide *m/z*	Fatty Acid	Tentative ID
654.36	d31:3	224.24	15:0 – H_2O	250.25	16:3	C15:0/16:3
		236.24	16:1 – H_2O	256.26	15:2 + H_2O	C16:1/15:2
		264.27	18:1 – H_2O	228.23	13:2 + H_2O	C18:1/13:2
668.54	d32:3	236.24	16:1 – H_2O	252.27	16:2	C16:1/16:2
		238.25	16:0 – H_2O	268.26	16:3 + H_2O	C16:0/16:3
		250.25	17:1 – H_2O	238.25	15:2	C17:1/15:2
		264.27	18:1 – H_2O	242.25	14:2	C18:1/14:2
670.56	d32:2	236.24	16:1 – H_2O	236.24	16:1 – H_2O	C16:1/16:1
		250.25	17:1 – H_2O	240.24	15:1	C17:1/15:1
		264.27	18:1 – H_2O			C18:1/14:1
672.37	d32:1	224.24	15:0 – H_2O	250.25	17:1	C15:0/17:1
		236.24	16:1 – H_2O	256.26	16:0	C16:1/16:0
		264.27	18:1 – H_2O			C18:1/14:0
674.48	d32:0 or d31:1–OH	236.24	16:1 – H_2O			C16:1/15:0–OH
		256.26	16:0	256.26	16:0	C16:0/16:0
		264.27	18:1 – H_2O			C18:1/13:0–OH
		278.28	19:1 – H_2O			C19:1/12:0–OH
682.56	d33:3	236.24	16:1 – H_2O	266.28	17:2	C16:1/17:2
		252.27	17:0 – H_2O	250.25	16:3	C17:0/16:3
		264.27	18:1 – H_2O	256.26	15:2 + H_2O	C18:1/15:2
686.51	d33:1	238.25	16:0 – H_2O	268.26	17:1	C16:0/17:1
		250.25	17:1 – H_2O	256.26	16:0	C17:1/16:0
		264.27	18:1 – H_2O			C18:1/15:0
698.66	d34:2	236.24	16:1 – H_2O	282.28	18:1	C16:1/18:1
		252.27	17:0 – H_2O	266.28	17:2	C17:0/17:2
		264.27	18:1 – H_2O	254.25	16:1	C18:1/16:1
700.58	d34:1	264.34	18:1 – H_2O	256.35	16:0	C18:1/16:0
702.50	d34:0 or d33:1–OH	240.10	15:1 – H_2O	300.06	18:0 – H + OH	C15:1/18:0–OH
		250.25	17:1 – H_2O	272.26	16:0 – H + OH	C17:1/16:0–OH
		256.26	16:0 – H_2O	284.29	18:0	C16:0/18:0
		264.27	18:1 – H_2O			C18:1/15:0–OH
710.59	d35:3	236.24	16:1 – H_2O			C16:1/19:2
		252.27	17:0 – H_2O	278.28	18:3	C17:0/18:3
		264.27	18:1 – H_2O	284.29	17:2 + H_2O	C18:1/17:2
712.57	d35:2	250.25	17:1 – H_2O	264.27	18:1 – H_2O	C17:1/18:1
		264.27	18:1 – H_2O	250.25	17:1 – H_2O	C18:1/17:1
		278.28	19:1 – H_2O			C19:1/16:1
714.59	d35:1	250.25	17:1 – H_2O	266.28	18:0 – H_2O	C17:1/18:0

		264.27	18:1 − H_2O	252.27	17:0 − H_2O	C18:1/17:0
		278.28	19:1 − H_2O	256.26	16:0	C19:1/16:0
716.41	d35:0 or d34:1−OH	236.24	16:1 − H_2O	300.29	18:0 − H + OH	C16:1/18:0−OH
		252.27	17:0 − H_2O	284.29	18:0	C17:0/18:0
		264.27	18:1 − H_2O	272.26	16:0 − H + OH	C18:1/16:0−OH
726.54	d36:2	252.27	17:0 − H_2O			C17:0/19:2
		264.27	18:1 − H_2O	282.28	18:1	C18:1/18:1
728.41	d36:1	236.24	16:1 − H_2O	312.33	20:0	C16:1/20:0
		252.27	17:0 − H_2O	278.29	19:1 − H_2O	C17:0/19:1
		264.27	18:1 − H_2O	284.29	18:0	C18:1/18:0
		292.30	20:1 − H_2O			C20:1/16:0
730.53	d36:0 or d35:1−OH	250.25	17:1 − H_2O			C17:1/18:0−OH
		252.27	17:0 − H_2O	298.55	19:0	C17:0/19:0
		264.27	18:1 − H_2O			C18:1/17:0−OH
		278.29	19:1 − H_2O			C19:1/16:0−OH
736.41	d37:4	224.24	15:0 − H_2O	314.28	22:4 − H_2O	C15:0/22:4
		236.24	16:1 − H_2O			C16:1/21:3
		262.25	18:2 − H_2O			C18:2/19:2
		264.27	18:1 − H_2O			C18:1/19:3
738.50	d37:3	224.24	15:0 − H_2O			C15:0/22:3
		236.24	16:1 − H_2O	340.36	21:2 + H_2O	C16:1/21:2
		264.27	18:1 − H_2O	312.33	19:2 + H_2O	C18:1/19:2
740.52	d37:2	236.24	16:1 − H_2O			C16:1/21:1
		264.27	18:1 − H_2O	278.28	19:1 − H_2O	C18:1/19:1
		278.28	19:1 − H_2O	264.27	18:1 − H_2O	C19:1/18:1
744.55	d37:0 or d36:1−OH	236.24	16:1 − H_2O			C16:1/20:0−OH
		252.27	17:0 − H_2O			C17:0/20:0
		264.27	18:1 − H_2O	300.29	18:0 − H + OH	C18:1/18:0−OH
752.63	d38:3	224.24	15:0 − H_2O			C15:0/23:3
		236.24	16:1 − H_2O	354.37	22:2 + H_2O	C16:1/22:2
		250.25	17:1 − H_2O	340.36	21:2 + H_2O	C17:1/21:2
		264.27	18:1 − H_2O	326.34	20:2 + H_2O	C18:1/20:2
754.53	d38:2	224.24	15:0 − H_2O			C15:0/23:2
		236.24	16:1 − H_2O	356.35	22:1 + H_2O	C16:1/22:1
		250.25	17:1 − H_2O			C17:1/21:1
		264.27	18:1 − H_2O			C18:1/20:1
756.55	d38:1	224.24	15:0 − H_2O			C15:0/23:1
		236.24	16:1 − H_2O	340.36	22:0	C16:1/22:0
		250.25	17:1 − H_2O			C17:1/21:0
		256.26	16:0			C16:0/22:1
		264.27	18:1 − H_2O	312.33	20:0	C18:1/20:0
766.53	d39:3	236.24	16:1 − H_2O	368.39	23:2 + H_2O	C16:1/23:2
		250.25	17:1 − H_2O	354.37	22:2 + H_2O	C17:1/22:2
		264.27	18:1 − H_2O	340.36	21:2 + H_2O	C18:1/21:2
768.55	d39:2	224.24	15:0 − H_2O	282.37	24:2 + H_2O	C15:0/24:2
		236.24	16:1 − H_2O	370.37	23:1 + H_2O	C16:1/23:1

		250.25	17:1 − H_2O	356.35	22:1 + H_2O	C17:1/22:1
		264.27	18:1 − H_2O			C18:1/21:1
770.57	d39:1	224.24	15:0 − H_2O			C15:024:0
		236.24	16:1 − H_2O	354.37	23:0	C16:1/23:0
		250.25	17:1 − H_2O	340.36	22:0	C17:1/22:0
		264.27	18:1 − H_2O	326.34	21:0	C18:1/21:0
772.58	d39:0 or d38:1−OH	224.24	15:0 − H_2O			C15:0/24:0
		236.24	16:1 − H_2O	356.35	22:0 − H + OH	C16:1/22:0−OH
		264.27	18:1 − H_2O			C18:1/20:0−OH
780.67	d40:3	236.24	16:1 − H_2O			C16:1/24:2
		250.25	17:1 − H_2O	368.39	23:2 + H_2O	C17:1/23:2
		252.27	17:0 − H_2O			C17:0/23:3
		264.27	18:1 − H_2O	354.37	22:2 + H_2O	C18:1/22:2
		278.28	19:1 − H_2O	340.36	21:2 + H_2O	C19:1/21:2
782.56	d40:2	224.24	15:0 − H_2O			C15:0/25:2
		236.24	16:1 − H_2O	384.38	24:1 + H_2O	C16:1/24:1
		250.25	17:1 − H_2O	270.37	23:1 + H_2O	C17:1/23:1
		264.27	18:1 − H_2O	356.35	22:1 + H_2O	C18:1/22:1
784.66	d40:1	236.40	16:1 − H_2O	368.50	24:0	C16:1/24:0
		264.34	18:1 − H_2O	340.36	22:0	C18:1/22:0
786.53	d40:0 or d39:1−H	224.24	15:0 − H_2O	382.36	25:0	C15:0/25:0
		236.24	16:1 − H_2O	370.37	23:0 − H + OH	C16:1/23:0−OH
		250.25	17:1 − H_2O	356.35	22:0 − H + OH	C17:1/22:0−OH
		264.27	18:1 − H_2O			C18:1/21:0−OH
794.56	d41:3	252.27	17:0 − H_2O			C17:0/24:3
		264.27	18:1 − H_2O	368.39	23:2 + H_2O	C18:1/23:2
		278.28	19:1 − H2O	354.37	22:2 + H_2O	C19:1/22:2
796.66	d41:2	252.27	17:0 − H_2O			C17:0/24:2
		264.27	18:1 − H_2O	352.36	23:1	C18:1/23:1
		278.28	19:1 − H_2O	356.35	22:1 + H_2O	C19:1/22:1
798.68	d41:1	250.33	17:1 − H_2O			C17:1/24:0
		264.40	18:1 − H_2O	354.60	23:0	C18:1/23:0
		278.35	19:1 − H_2O			C19:1/22:0
800.58	d41:0 or d40:1−OH	224.24	15:0 − H_2O			C15:0/26:0
		236.24	16:1 − H_2O	384.38	24:0 − H + OH	C16:1/24:0−OH
		250.25	17:1 − H_2O	370.37	23:0 − H + OH	C17:1/23:0−OH
		252.27	17:0 − H_2O			C17:0/24:0
		256.26	16:0			C16:0/25:0
		264.27	18:1 − H_2O	356.35	22:0 − H + OH	C18:1/22:0−OH
		278.29	19:1 − H_2O			C19:1/21:0−OH
		338.34	22:1	300.29	18:0 − H + OH	C22:1/18:0−OH
		366.37	24:1	272.26	16:0 − H + OH	C24:1/16:0−OH
810.68	d42:2	236.24	16:1 − H_2O	394.36	26:1	C16:1/26:1
		252.27	17:0 − H_2O	396.39	25:2 + H_2O	C17:0/25:2
		264.27	18:1 − H_2O	366.37	24:1	C18:1/24:1
		278.28	19:1 − H_2O	352.36	23:1	C19:1/23:1

		292.30	20:1 − H_2O			C20:1/22:1
812.54	d42:1	236.24	16:1 − H_2O	378.58	26:0 − H_2O	C16:1/26:0
		250.25	17:1 − H_2O			C17:1/25:0
		252.27	17:0 − H_2O			C17:0/25:1
		264.27	18:1 − H_2O	368.39	24:0	C18:1/24:0
		278.28	19:1 − H_2O	354.37	23:0	C19:1/23:0
		292.30	20:1 − H_2O			C20:1/22:0
814.60	d42:0 or d41:1–OH	236.24	16:1 − H_2O			C16:1/25:0–OH
		252.27	17:0 − H_2O			C17:0/25:0
		264.27	18:1 − H_2O	384.38	24:0 − H + OH	C18:1/24:0–OH
		278.28	19:1 − H_2O	370.37	23:0 − H + OH	C19:1/23:0–OH
824.76	d43:2	252.27	17:0 − H_2O			C17:0/26:2
		264.27	18:1 − H_2O	380.39	25:1	C18:1/25:1
		278.28	19:1 − H_2O	366.37	24:1	C19:1/24:1
		292.30	20:1 − H_2O			C20:1/23:1
826.60	d43:1	250.25	17:1 − H_2O			C17:1/26:0
		252.27	17:0 − H_2O			C17:0/26:1
		264.27	18:1 − H_2O	382.40	25:0	C18:1/25:0
		278.28	19:1 − H_2O	368.39	24:0	C19:1/24:0
		292.30	20:1 − H_2O	354.37	23:0	C20:1/23:0
828.65	d43:0 or d42:1–OH	236.24	16:1 − H_2O			C16:1/26:0–OH
		250.25	17:1 − H_2O			C17:1/25:0–OH
		252.27	17:0 − H_2O	396.38	26:0	C17:0/26:0
		264.27	18:1 − H_2O	384.38	24:0 − H + OH	C18:1/24:0–OH
		278.28	19:1 − H_2O	370.37	23:0 − H + OH	C19:1/23:0–OH
		292.30	20:1 − H_2O			C20:1/22:0–OH
		366.37	24:1 − H_2O	300.29	18:0 − H + OH	C24:1/18:0–OH
838.78	d44:2	252.27	17:0 − H_2O			C17:0/27:2
		264.27	18:1 − H_2O	412.42	26:1 + H_2O	C18:1/26:1
		278.28	19:1 − H_2O			C19:1/25:1
		292.30	20:1 − H_2O			C20:1/24:1
840.64	d44:1	252.27	17:0 − H_2O			C17:0/27:1
		264.27	18:1 − H_2O			C18:1/26:0
		278.30	19:1 − H_2O	400.98	25:0 + H_2O	C19:1/25:0
		292.30	20:1 − H_2O	368.39	24:0	C20:1/24:0

Table S8. List of LacCer molecular species found in MFGM Lipid 100 with their tentative identification based on observed evidence.

m/z	d-value	Backbone *m/z*	Sphingoid Base	Fatty Amide *m/z*	Fatty Acid	Tentative ID
862.62	d34:1	236.24	16:1 − H_2O			C16:1/18:0
		252.27	17:0 − H_2O			C17:0/17:1
		264.27	18:1 − H_2O	256.26	16:0	C18:1/16:0
864.63	d34:0 or d33:1–OH	256.26	16:0	284.29	18:0	C16:0/18:0
		264.27	18:1 − H_2O			C18:1/15:0–OH
		284.29	18:0	256.26	16:0	C18:0/16:0

876.59	d35:1	250.25	17:1 − H_2O	284.29	18:0	C17:1/18:0
		264.27	18:1 − H_2O	270.28	17:0	C18:1/17:0
		278.28	19:1 − H_2O	274.07	16:0 + H_2O	C19:1/16:0
890.65	d36:1	236.24	16:1 − H_2O	312.32	20:0	C16:1/20:0
		252.27	17:0 − H_2O			C17:0/19:1
		264.27	18:1 − H_2O	284.29	18:0	C18:1/18:0
900.55	d37:3	224.24	15:0 − H_2O			C15:0/22:3
		236.24	16:1 − H_2O	340.36	21:2 + H_2O	C16:1/21:2
		264.27	18:1 − H_2O			C18:1/19:2
914.69	d38:3	224.24	15:0 − H_2O			C15:0/23:3
		236.24	16:1 − H_2O	354.37	22:2 + H_2O	C16:1/22:2
		250.25	17:1 − H_2O	340.36	21:2 + H_2O	C17:1/21:2
		264.27	18:1 − H_2O	326.34	20:2 + H_2O	C18:1/20:2
916.59	d38:2	236.24	16:1 − H_2O			C16:1/22:1
		256.26	16:0	318.08	22:2 − H_2O	C16:0/22:2
		264.27	18:1 − H_2O			C18:1/20:1
918.69	d38:1	224.38	15:0 − H_2O			C15:0/23:1
		236.27	16:1 − H_2O	340.63	22:0	C16:1/22:0
		238.52	16:0 − H_2O			C16:0/22:1
		264.52	18:1 − H_2O			C18:1/20:0
		266.72	18:0 − H_2O			C18:0/20:1
920.82	d38:0 or d37:1–OH	236.24	16:1 − H_2O			C16:1/21:0–OH
		256.26	16:0	340.36	22:0	C16:0/22:0
		264.27	18:1 − H_2O			C18:1/19:0–OH
		284.29	18:0			C18:0/20:0
928.70	d39:3	224.24	15:0 − H_2O			C15:0/24:3
		236.24	16:1 − H_2O	368.39	23:2 + H_2O	C16:1/23:2
		250.25	17:1 − H_2O	354.37	22:2 + H_2O	C17:1/22:2
		264.27	18:1 − H_2O	340.36	21:2 + H_2O	C18:1/21:2
932.70	d39:1	236.36	16:1 − H_2O			C16:1/23:0
		264.22	18:1 − H_2O	326.59	21:0	C18:1/21:0
		268.24	17:1	340.68	22:0	C17:1/22:0
934.71	d39:0 or d38:1–OH	236.24	16:1 − H_2O			C16:1/22:0–OH
		256.26	16:0	354.37	23:0	C16:0/23:0
		274.27	16:0 + H_2O	354.37	22:1 − H + OH	C16:0/22:1–OH
942.72	d40:3	236.24	16:1 − H_2O			C16:1/24:2
		250.25	17:1 − H_2O	368.39	23:2 + H_2O	C17:1/23:2
		264.27	18:1 − H_2O	354.37	22:2 + H_2O	C18:1/22:2
946.72	d40:1	236.30	16:1 − H_2O	368.76	24:0	C16:1/24:0
		250.32	17:1 − H_2O			C17:1/23:0
		264.37	18:1 − H_2O	340.36	22:0	C18:1/22:0
		278.56	19:1 − H_2O			C19:1/21:0
948.73	d40:0 or d39:1–OH	236.24	16:1 − H_2O			C16:1/23:0–OH
		250.25	17:1 − H_2O			C17:1/22:0–OH
		256.26	16:0	368.39	24:0	C16:0/24:0
		264.27	18:1 − H_2O			C18:1/21:0–OH

		284.29	18:0	340.36	22:0	C18:0/22:0
954.76	d41:4	254.27	16:1			C16:1/25:3
960.73	d41:1	250.07	17:1 − H_2O	354.46	23:0	C17:1/24:0
		252.36	17:0 − H_2O			C17:0/24:1
		264.37	18:1 − H_2O			C18:1/23:0
		278.23	19:1 − H_2O			C19:1/22:0
		292.36	20:1 − H_2O			C20:1/21:0
974.75	d42:1	250.31	17:1 − H_2O	350.42	24:0 − H_2O	C17:1/25:0
		252.50	17:0 − H_2O			C17:0/25:1
		264.17	18:1 − H_2O			C18:1/24:0
		278.25	19:1 − H_2O			C19:1/23:0
986.66	d43:2	252.27	17:0 − H_2O	392.39	26:2	C17:0/26:2
		264.27	18:1 − H_2O	380.39	25:1	C18:1/25:1
		278.28	19:1 − H_2O			C19:1/24:1
		292.30	20:1 − H_2O			C20:1/23:1
988.59	d43:1	252.27	17:0 − H_2O			C17:0/26:1
		264.27	18:1 − H_2O	382.40	25:0	C18:1/25:0
		278.28	19:1 − H_2O	386.12	24:0 + H_2O	C19:1/24:0
		292.30	20:1 − H_2O	354.37	23:0	C20:1/23:0
992.76	d44:6	262.25	18:2 − H_2O			C18:2/26:4
		264.27	18:1 − H_2O	386.12	26:5	C18:1/26:5
		300.29	21:4 − H_2O	368.39	23:2 + H_2O	C21:4/23:2
		318.3	22:2 − H_2O	332.32	22:4	C22:2/22:4
1002.77	d44:1	252.27	17:0 − H_2O	396.42	26:0	C17:0/27:1
		264.27	18:1 − H_2O			C18:1/26:0
		278.28	19:1 − H_2O			C19:1/25:0
		292.30	20:1 − H_2O	368.39	24:0	C20:1/24:0

Table S9. Responses of GalCer 18:1/18:0 and GluCer 18:1/18:0 at the same molar concentrations and their comparisons.

	GalCer 18:1/18:0 Area	GluCer 18:1/18:0 Area	Ratio
12.5 μM	8.29E + 07	7.98E + 07	104%
25 μM	2.03E + 08	1.90E + 08	107%
50 μM	2.89E + 08	3.17E + 08	91%
100 μM	4.56E + 08	4.62E + 08	99%
200 μM	6.32E + 08	6.49E + 08	97%
